# Selection of Potent Inhibitors of Soluble Epoxide Hydrolase for Usage in Veterinary Medicine

**DOI:** 10.3389/fvets.2020.00580

**Published:** 2020-08-26

**Authors:** Diyala S. Shihadih, Todd R. Harris, Sean D. Kodani, Sung-Hee Hwang, Kin Sing Stephen Lee, Vengai Mavangira, Briana Hamamoto, Alonso Guedes, Bruce D. Hammock, Christophe Morisseau

**Affiliations:** ^1^Department of Entomology and Nematology, U.C. Davis Comprehensive Cancer Center, University of California, Davis, Davis, CA, United States; ^2^Department of Pharmacology and Toxicology and Department of Chemistry, Michigan State University, East Lansing, MI, United States; ^3^Department of Large Animal Clinical Sciences, College of Veterinary Medicine, Michigan State University, East Lansing, MI, United States; ^4^Department of Surgical and Radiological Sciences, School of Veterinary Medicine, University of California, Davis, Davis, CA, United States; ^5^Department of Veterinary Clinical Sciences, College of Veterinary Medicine, University of Minnesota, St Paul, MN, United States

**Keywords:** epoxyeicosatrienoic acids, dog, cat, horse, pain

## Abstract

The veterinary pharmacopeia available to treat pain and inflammation is limited in number, target of action and efficacy. Inhibitors of soluble epoxide hydrolase (sEH) are a new class of anti-inflammatory, pro-resolving and analgesic drugs being tested in humans that have demonstrated efficacy in laboratory animals. They block the hydrolysis, and thus, increase endogenous concentrations of analgesic and anti-inflammatory signaling molecules called epoxy-fatty acids. Here, we screened a library of 2,300 inhibitors of the sEH human against partially purified feline, canine and equine hepatic sEH to identify inhibitors that are broadly potent among species. Six very potent sEH inhibitors (IC_50_ < 1 nM for each enzyme tested) were identified. Their microsomal stability was then measured in hepatic extracts from cat, dog and horse, as well as their solubility in solvents suitable for the formulation of drugs. The *trans*-4-{4-[3-(4-trifluoromethoxy-phenyl)-ureido]-cyclohexyloxy}-benzoic acid (t-TUCB, **1,728**) appears to be the best compromise between stability and potency across species. Thus, it was selected for further testing in veterinary clinical trials of pain and inflammation in animals.

## Introduction

Pain management is central to the effective treatment of both human and veterinary patients. Pain control improves both the outcomes of disease and the quality of life (1). Currently, the main class of veterinary drugs used to reduce pain and inflammation in animals, including horses, cats, and dogs, are non-steroidal anti-inflammatory drugs (NSAIDs), cyclooxygenase inhibitors (COXIBs) and corticosteroids. As well as being marginally effective in some species, these categories of pharmacological drugs have dose dependent deleterious side effects that limits their use, especially in the management of chronic pain (2). For example, chronic laminitis in horses is an extremely painful condition that too often leads to euthanasia due to the inability of currently available analgesics to adequately control pain (3). Osteoarthritis is another chronic painful condition, especially in elderly cats and dogs, that can be difficult to manage with currently available analgesics. It cannot be treated with NSAIDs or COXIBs chronically because of adverse side effects, such as liver toxicity and gastric ulceration (4, 5). These challenges underline the urgent need for novel pain medication in veterinary medicine.

In vertebrates, the arachidonic acid (AA) cascade plays a major role in the regulation of multiple biological processes, including pain and inflammation (6). While the cyclooxygenase (COX) and lipoxygenase (LOX) branches of the cascade are targets of many pharmaceuticals on the market, including NSAIDs and COXIBs, the CYP450 branch of the cascade has yet to be targeted in the same manner. Within the P450 branch, epoxide metabolites of AA, called epoxy-eicosatrienoic acids (EETs), have anti-inflammatory and analgesic effects (7). However, the endogenous hydrolysis of the EETs to their corresponding diols by soluble epoxide hydrolase (sEH) reduces their biological activity (8). Both *in vitro* and *in vivo* studies have demonstrated that the modulation of chronic inflammation and neuronal pain by EETs is inversely dependent on the extent of their hydrolysis by sEH (7–9). Thus, the use of sEH inhibitors to maintain high *in vivo* levels of EETs is a promising pharmacological approach to treat pain and inflammation (10). Consequently, sEH inhibitors are in development for the treatment of neuropathic pain in human patients (9). In addition to their use in humans, sEH inhibitors may become suitable analgesics for use in veterinary medicine (11–14).

Preliminary data obtained with compounds optimized for the human sEH show that sEH inhibition has the potential to reduce pain in dogs with osteoarthritis (14), and horses with chronic laminitis (12). However, sEH inhibitors optimized for use in primates and rodents are typically not sufficiently potent inhibitors or are metabolized too rapidly for chronic use in other mammals (15). Therefore, a library of 2,300 sEH inhibitors developed for the human enzyme was screened with partially purified feline, canine and equine hepatic sEH to identify inhibitors that are broadly potent among species. In addition, microsomal stability of potent compounds was tested, as well as the solubility of the selected inhibitor in solvents typically used in the formulation of drugs.

## Materials and Methods

### Chemicals

(3-Phenyl-oxiranyl)-acetic acid cyano-(6-methoxy-naphthalen -2-yl)-methyl ester (PHOME), cyano(6-methoxynaphthalen-2-yl)methyl phenyl butyrate (CMNPB), cyano(6-methoxynaphthalen-2-yl)methyl ((3-phenyloxiran-2-yl)methyl) carbonate (CMNPC), and 4-((4-(3-(4-(trifluoromethoxy)phenyl)ureido)cyclohexyl)oxy)benzoic acid (*t*-TUCB) were synthesized previously in the laboratory (16, 17). Paraoxon and other chemicals were obtained from Sigma or Fisher Scientific and used without further purification. The library of human sEH inhibitors was previously prepared in the laboratory (18). The library was prepared in 2-mL deep-well polypropylene 96-well assay plates. Every compound was prepared at 10 mM in dimethyl sulfoxide (DMSO). Only compounds completely soluble at 10 mM in DMSO were included in the library. In each plate, the wells in the first column contained only DMSO to serve as controls. In the remainder of the plate, we dispensed one compound per well, with 88 compounds total per plate. Twenty-six plates were created with different chemicals for a total of 2,288 compounds. The plates were tightly sealed with EVA copolymer sealing mats. The plates were then sealed in a 2-mil. thick plastic bag, to avoid condensation, and stored at −20°C until use. Upon usage, the plates were allowed to warm-up to room temperature overnight before removal from the plastic bag. Using a robotic pipetting station (Quadra 96 – 96 well-automated pipettor; Tomtec, Hamden, CT), each well was first mixed and the compound solutions were diluted 10-fold in DMSO (down to 1 mM) and then further diluted in the appropriate buffer and transferred to black 96-well plates.

### Tissue Samples and Partial Purification

Fresh liver tissues samples (5–30 g) were collected promptly after death from healthy adult cat, dog and horse euthanized for reasons unrelated to this study, and whose bodies were given to science by the owners in line with federal regulations and protocol approved by the Institutional Animal Care and Use Committee at University of California Davis. Similarly, tissues from the other animals tested herein were collected from healthy animals immediately after being slaughter at the university abattoir. Because no animals were directly used or killed for this study, sample collections were exempted from approval by the Institutional Animal Care and Use Committee of the University of California Davis and Michigan State University. Samples were taken from livers that upon gross evaluation did not display any sign of clinical disease. The tissues were flash-frozen in liquid nitrogen following removal, and then kept at −80°C until used. After thawing on ice, the samples were homogenized in chilled sodium phosphate buffer (20 mM pH 7.4) containing 5 mM EDTA, 1 mM DTT and 1 mM PMSF (tissue weight:buffer volume ratio 1:4). These homogenates were centrifuged at 100,000 g for 60 min at 4°C. The supernatant (the cytosolic fraction) was separated from the pellet, aliquoted and flash frozen in liquid nitrogen, then kept frozen at −80°C until usage.

The sEH activity from the cat, dog and horse liver was further purified to eliminate contaminating esterase activity that could interfere with the screening fluorescent assay (16). The cytosolic fraction was loaded on a Q-Sepharose column (100 mL) equilibrated with sodium phosphate buffer (20 mM pH 7.4). After washing of the bound proteins, the sEH activity was eluted with a step gradient of NaCl (0–1 M). The fractions containing the sEH activity were pooled and concentrated using a Centriprep-30. The concentrates were aliquoted and flash frozen, then kept frozen at −80°C until usage. To ensure total elimination of residual esterase activity, the purified enzymes were further treated with 100 μM of Paraoxon, a non-selective pan-esterase inhibitor (19) that does not inhibit sEH (18). Protein concentration was quantified using the Pierce BCA assay (Pierce, Rockford, IL), using Fraction V bovine serum albumin (BSA) as the calibrating standard.

### Activity Measurement in Extracts

The sEH activity was measured using [^3^H]-labeled *trans*-diphenyl propene oxide ([^3^H]*t*-DPPO) as substrate (20), while the esterase activity was measured using CMNPB as substrate (21). For both assays, the cellular extracts were diluted in sodium phosphate buffer (0.1 M pH 7.4) containing 0.1 mg/mL of BSA. The activities were measured with a final substrates concentration of 50 μM, for 5–10 min at 37°C).

### Screening of the Library of Inhibitors

Primary screening of the library was performed with an inhibitor concentration of 100 nM using PHOME as substrate (22). To black 96-well plates containing 20 μL of 1 μM of the test-compound solutions, 150 μL of sodium phosphate buffer (0.1 M pH 7.4) containing 0.1 mg/mL of BSA were added in wells A1 to D1 (these four wells served as background control), and 150 μL of the enzyme diluted in the same buffer ([protein]_final_ for horse sEH: 17 μg/mL; cat sEH: 99 μg/mL; dog sEH: 208 μg/mL) were added to the rest of the plate (wells E1 to H1 served as full activity control). The plates were then mixed and incubated at 30°C for 5 min. Across each plate, 30 μL of the working substrate solution (270 μL of 5 mM PHOME solution in DMSO diluted with 3,680 μL of buffer) were added quickly to yield [S]_final_: 50 μM. Each plate was further incubated at room temperature (horse sEH: 60 min; cat sEH: 45 min; dog sEH: 30 min). The formation of the fluorescent product, 6-methoxynaphthaldehyde (λ_excitation_ = 330 nm, λ_emission_ = 465 nm) was measured with a Spectramax M2 spectrophotometer (Molecular Devices, Sunnyvale, CA). To ensure the validity of the high throughput screen (HTS), results from the background control wells (A1 to D1) and full activity control wells (E1 to H1) were used to calculate the signal to noise ratio and Z′ factor (23).

Compounds that gave more than 90% inhibition for one of the three enzymes tested during the primary screen were collected and distributed in five new 96-well plates (88 compounds per plate, 390 compounds in total). The library of selected compounds was screened with [I] = 100 nM using PHOME as substrate, in conditions similar to the primary screening with the exception of the plate reading to identify possible false positive. Following substrate addition, the activity was immediately measured at 30°C kinetically for 15 min by measuring the fluorescent signal (λ_excitation_ = 330 nm, λ_emission_ = 465 nm, 30°C) every 30 s.

### Determination of sEH Inhibition Potency (IC_50_)

The IC_50_ is the concentration of an inhibitor that reduces the sEH activity by 50%. The IC_50_ values reported herein were determined using either a fluorescent based assay (CMNPC as substrate) for purified enzyme preparations or a radioactive based assay ([^3^H]*t*-DPPO as substrate) for crude enzyme preparations (20). The fluorescent assay was used with the purified proteins. The enzymes ([E]_final_ for horse sEH: 17 μg/mL; cat sEH: 99 μg/mL; dog sEH: 208 μg/mL) were incubated at 30°C with the inhibitors ([I]_final_ = 0.4–1,000 nM) for 5 min in 100 mM sodium phosphate buffer (200 μL pH 7.4) containing 0.1 mg/mL of BSA and 1% of DMSO. The substrate (CMNPC) was then added ([S]_final_ = 5 μM). Activity was assessed by measuring the appearance of the fluorescent 6-methoxynaphthaldehyde product (λ_ex_ = 330 nm, λ_em_ = 465 nm) every 30 s for 10 min at 30°C. The radioactive fluorescent assay was used with the liver crude extracts. Extracts were incubated with inhibitors ([I]_final_ = 1–1,000 nM) at 30° C for 5 min in sodium phosphate buffer (pH 7.4) containing 0.1 mg/mL of BSA and 1% of DMSO, prior to substrate introduction ([^3^H]*t*-DPPO [S]_final_: 50 μM; ~12,000 dpm/assay). The enzymes were incubated at 30°C for 10 min, and the reaction was quenched by the addition of 60 μL methanol and 200 μL isooctane, which extracts the remaining epoxide from the aqueous phase. The activity was followed by measuring the quantity of radioactive diol formed in the aqueous phase using a liquid scintillation counter (Tri-Carb 2810TR, Perkin Elmer, Waltham, MA). Under the conditions used, rates were linear with both time and enzyme concentration and resulted in at least 5% but no more than 30% hydrolysis of the substrate. Assays were performed in triplicate. The IC_50_ values were calculated from at least five different concentrations, each in triplicate, with at least two on either side of 50% activity mark.

### Microsomal Stability

The stability of the sEH inhibitors was determined using liver microsomes as described (24). The inhibitors ([I]_final_ = 1 μM) were added to a suspension of liver microsomes ([protein]_final_= 1 mg/mL) in potassium phosphate buffer (0.1M pH7.4) containing 3 mM MgCl_2_ and 1 mM EDTA. After 5 min incubation at 37°C, the reaction was started by the addition of an NADPH generating system (buffer was added in the control tubes) (24). After 30 min at 37°C, the reaction was stopped by the addition of one volume of methanol containing CUDA (200 nM) as surrogate. The amount of remaining inhibitor was determined by LC/MS/MS. The stability is reported as a percentage of compound remaining after 30 min in these conditions. Results are triplicate average ± standard deviation.

Mass spectrometry analyses were performed using a Waters Quattro Premier triple quadrupole tandem mass spectrometer (Micromass, Manchester, UK) interfaced to an electrospray ionization (ESI) source. The MS was coupled with a Waters Acquity UPLC (Waters, Milford, MA, USA). A Varian Pursuit5 C18 RP HPLC column (150 mm × 2.1 mm, particle size 5 μm) was used to separate the analytes. The ESI was performed following HPLC in the positive mode at 2.51 kV capillary voltage. The source and the desolvation temperatures were set at 120 and 300°C, respectively. Cone gas (N_2_) and desolvation gas (N_2_) were maintained at flow rates of 10 and 700 L/h, respectively. Dwell time was set to 0.1 s. A regression curve for each compound was obtained from at least six different concentrations of standard stock solutions (*R*^2^ > 0.99).

### Solubility of Inhibitors in Different Solvents

Solubility was determined by shaking vigorously excess *t*-TUCB (0.25–2 grams) in 10 mL of 11 different solvents at 30 or 40°C for 24 h in glass vials. The un-dissolved compounds were filtered through a 0.22 μm centrifuge filter at 30 or 40°C and the supernatant further diluted 10-fold with methanol. The concentration of *t*-TUCB in the samples was determined by LC/MS/MS analysis as previously described (15).

## Results

### Partial Purification of Animal sEH

Liver represents an excellent source of sEH because it is a large organ and the sEH activity is the highest in this organ across species (25). Liver cytosolic fractions are commonly used to measure sEH activity with the radioactive substrate [^3^H]*t*-DPPO (20). However, this substrate is not appropriate for an HTS of a library of compounds due to the low throughput of the technique. Unfortunately, the sEH fluorescent substrate developed for the HTS, PHOME, is not suitable for usage with crude lysate since it can also be hydrolyzed rapidly by esterases and can react chemically and enzymatically with glutathione (22). The glutathione and these enzymes are highly abundant in the liver, thus yielding a very high background that cannot be eliminated totally with chemical treatment. Esterase activity was measured using CMNPB, a fluorescent substrate that is structurally similar to the EH substrate but does not contain the epoxide moiety. To eliminate the glutathione cofactor and to reduce the esterase activity, a partial purification of the liver sEH was performed. A simple gravity anionic exchange column allows the removal of all the glutathione and over 90% of the unwanted esterase activity while retaining more than 67% of the targeted sEH activity, resulting in a 6.5- to 9.9-fold increase in specific activity (Table 1). The remaining small amount of esterase activity was eliminated totally with treatment of the partially purified preparation with paraoxon, a non-selective serine esterase inhibitor (19) that does not inhibit sEH. These preparations were used for the screening of the library of compounds.

**Table 1:** Partial purification of sEH activity from horse, cat and dog liver to remove esterase activity.

		**Soluble epoxide hydrolase activity**		**Esterase activity**	
**Fraction**	**Total protein (mg)**	**Total act. (nmol.min^**−1**^) (Yield%)**	**Spec. act. (nmol.min^**−1**^. mg^**−1**^)**	**Total act. (nmol.min^**−1**^) (Yield%)**	**Spec. act. (nmol.min^**−1**^.mg^**−1**^)**
**Horse from 30.6 g of liver**					
Cytosol	3,500	83,160 (100)	24	452,120 (100)	129
Final prep.	360	55,720 (67)	155	4,940 (1)	14
**Cat from 15.9 g of liver**					
Cytosol	2,240	18,250 (100)	8.1	60,190 (100)	27
Final prep.	198	15,850 (87)	80.0	3,020 (5.1)	15
**Dog from 25.1 grams of liver**					
Cytosol	2,630	6,990 (100)	2.7	268,260 (100)	120
Final prep.	234	5,440 (78)	23.2	20,600 (8)	88

### Library Screening and Selection of Potent Compounds

The compound library was initially screened using an endpoint assay. For all three species, the amount of enzyme and time of incubation were optimized to ensure linear substrate hydrolysis over the period tested, while limiting the total substrate conversion to under 20% as recommended in the literature (22). Using the optimized conditions described in the method section, a 26-plate library (2,288 compounds in total) was screened at a final inhibitor concentration of 100 nM. To determine the appropriateness and robustness of the assays for HTS, the Z′ factor and S/N were calculated (Table 2) (23). For all three enzymes, Z' values were between 0.7 and 0.8 and the S/N value > 80. This demonstrated that the assay has a large window between samples and blank signals and thereby confirms that it was suitable for HTS. For each enzyme tested, between 4 and 15% of compounds in the targeted library were positive hits (> 90% inhibition). In total, 390 compounds were found to strongly inhibit sEH of at least one of the animal species tested. Occasionally, false positives can occur when the tested inhibitor quenches the fluorescence of the enzymatic product. Thus, these positive hits were reorganized in five plates, and the potency of this subset of compounds was tested against the three enzymes at 100 nM. The plates were read in a kinetic mode to eliminate false positives. Again, both the Z' and S/N factors were high for all three enzymes (Table 2), underlying the robustness of the assay. For most of the compounds tested, the potency of the primary screening was confirmed in the secondary screening (Table S1) indicating the accuracy of the primary screening.

**Table 2:** Screening assay performances.

**Assay**	**Horse sEH**			**Cat sEH**			**Dog sEH**		
	**Z'**	**S/N**	**# of hits**	**Z'**	**S/N**	**# of hits**	**Z'**	**S/N**	**# of hits**
Primary screen (end point) 26 plates	0.73 ± 0.06	101 ± 57	354	0.78 ± 0.09	102 ± 79	196	0.81 ± 0.07	89 ± 50	88
Secondary screen (kinetic mode) 5 plates	0.75 ± 0.04	96 ± 24	309	0.79 ± 0.06	60 ± 21	188	0.75 ± 0.05	39 ± 14	66
% false positive			13			4			25

*Results are average ± SD (primary screen n = 26; secondary screen n = 5)*.

Narrowing the focus to compounds that inhibit sEH in all three species, inhibitors yielding at least 80% inhibition for each enzyme were selected, and their inhibitory potencies (IC_50_) were determined (Table S2). Consistent with previous observations, the most potent inhibitors across species (Table S2) generally contained a central urea pharmacophore (17). This is due to the relatively strong H-bonding interaction between this pharmacophore and the enzyme active site residues that are conserved across species. On one side of the urea functional group, the presence of an adamantyl or substituted phenyl group yielded a potent inhibitor for all three enzymes. On the opposite side of the urea, alkyl or cyclohexyl were preferred, while phenyl or piperidine groups did not yield potent inhibitors of any of the enzymes tested. The six most potent inhibitors for all three animal species were selected for further testing (Table 3).

**Table 3:** Inhibition potency of selected compounds for the horse, cat, and dog sEH.

**EHI #**	**Structure**	**Horse sEH**	**Cat sEH IC_**50**_ (nM)**	**Dog sEH**
**700**	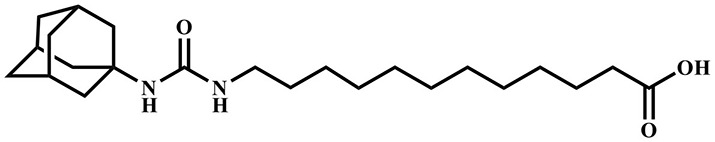	0.4 ± 0.02	0.4 ± 0.01	0.4 ± 0.01
**1,471**	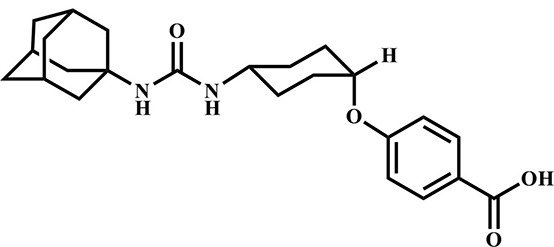	0.4 ± 0.01	1.0 ± 0.08	0.4 ± 0.01
**1,675**	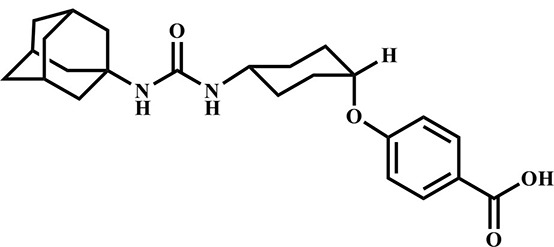	0.4 ± 0.01	0.4 ± 0.01	0.5 ± 0.03
**1,707**	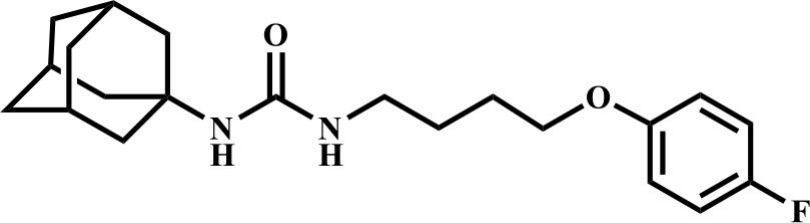	0.5 ± 0.03	0.5 ± 0.02	0.9 ± 0.1
**1,728**	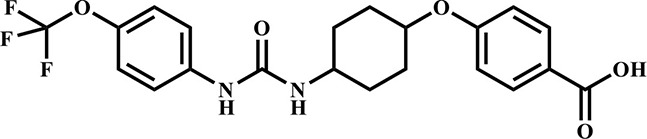	0.5 ± 0.02	0.4 ± 0.01	0.9 ± 0.05
**1,845**	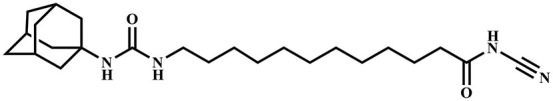	0.4 ± 0.01	0.4 ± 0.01	0.6 ± 0.04

*IC_50_s were determined using CMNPC ([S]_final_ = 5 μM) as substrate (16). Results are average ± standard deviation (n = 3)*.

Since sEH inhibitors could also be beneficial to treat animals other than horses, cats and dogs, especially farm animals, the potency of the selected six inhibitors was tested against the sEH activity of several common animals (Table 4) and compared to **1,770** (a.k.a. TPPU) a compound with demonstrated efficacy in rodent models. Except for **1,845**, the selected compounds were potent inhibitors of the sEH from the other tested species and so are potentially useful in the treatment of diverse mammalian species.

**Table 4:** Inhibition potency of the selected compounds for the sEH from other animals of veterinary medicine importance.

	**sEH activity from liver cytosol of**						
	**Mouse**	**Rat**	**Hamster**	**Rabbit**	**Pig**	**Cow**	**Sheep**
**#**	**IC**_**50**_ (nM)^*^						
**700**	10	11	5	20	11	6	3
**1,471**	8	8	2	4	8	2	5
**1,675**	4	7	5	2	12	2	4
**1,707**	10	7	2	6	6	7	2
**1,728**	11	6	5	2	9	2	6
**1,845**	17	12	34	45	81	11	7
**1,770**	8	14	25	275	75	210	44
[^3^H]*t*-DPPO activity (nmol.min^−1^.mg^−1^)	45 ± 6^#^	3.5 ± 1.0	12 ± 2	22 ± 3	4.6 ± 0.9	12 ± 1	25 ± 3

* *IC_50_s were determined using [^3^H]t-DPPO ([S]_final_ = 5 μM) as substrate (20)*.

# *Average ± SD (n ≥ 3)*.

### cLogP, Microsomal Stability, and Solubility

In addition to their on-target potency, inhibitors also need to be soluble and metabolically stable to be clinically useful. cLogP, a measure of both solubility and bioavailability, and microsomal stability, an *in vitro* measure of metabolism, were previously shown to influence greatly the *in vivo* potency of sEH inhibitors in rodents (26). The cLogP, and microsomal stability were determined for all six selected compounds (Table 5). The cLogPs were calculated using ChemBioDraw (v.16.0, CambridgeSoft). In their neutral form, all inhibitors have a cLogP above 4.5, suggesting limited solubility and possibly bioavailability (27). For **700** (a.k.a. AUDA), **1,471** (a.k.a. t-AUCB), **1,675**, and **1,728** (a.k.a. t-TUCB), the charged form (a.k.a. base form) of the acid function yields a significantly lower cLogP. This suggests that water solubility could be greatly increased in neutral to basic solutions.

**Table 5:** cLogP and stability of selected compounds.

	**Stability (% without microsomes)**^**a**^						**cLogP**
	**Horse liver microsomes**		**Cat liver microsomes**		**Dog liver microsomes**		
**NADPH**	**–**	**+**	**–**	**+**	**–**	**+**	
**700**	114 ± 15	96 ± 9	105 ± 11	28 ± 3	94 ± 6	66 ± 9	5.98 −1.14^b^
**1,471**	101 ± 15	97 ± 8	96 ± 7	77 ± 5	105 ± 14	102 ± 6	4.84 0.62^b^
**1,675**	104 ± 1	82 ± 18	70 ± 9	5 ± 3	96 ± 10	85 ± 9	4.84 0.62^b^
**1,707**	98.±4	1.6 ± 0.2	101 ± 14	5 ± 1	87 ± 17	8 ± 3	4.81
**1,728**	108 ± 10	89 ± 3	103 ± 8	101 ± 3	104 ± 3	100 ± 3	5.04 0.82
**1,845**	67 ± 3	28 ± 9	5 ± 1	9 ± 2	84 ± 3	34 ± 11	4.58

a *Stability measured with a liver microsome fraction (1 mg/mL) in the presence and absence of NADPH at 37°C for 30 min. Results presented as the relative amount of compound remaining measured by LC/MS. Results are average ± standard deviation (n = 3)*.

b *Base form*.

The stability of the six inhibitors (1 μM) in equine, feline and canine liver microsomes (1 mg/mL) was measured in the presence and absence of NADPH. After a 30-min incubation at 37°C, the amount of remaining inhibitor was quantified using mass spectrometry (Table 5) (28). The stability of the compounds varied widely based on structure and species. Even in the absence of NADPH, **1,845** was not stable, suggesting a non-P450 metabolism. It was metabolized faster in the cat than in the horse and dog. Because **1,845** contains an amide, its rapid metabolism probably resulted from the action of amidases that are well-known to be present in liver microsomes (29, 30). In dog and horse, **1,845** compound was further metabolized in the presence of NADPH underlying its overall instability, thus limiting its potential usage *in vivo*. While the other compounds were stable in absence of NADPH, their stability varied when it was added to the microsomes. While **1,707** was metabolized rapidly by the P450 s of all three species, **700**, and **1,675** were quickly transformed by the cat enzymes only, while **1,471** metabolism was slower. **1,728** was the most stable compound for all three species tested.

Besides having adequate potency, availability and stability, a good drug must also be amenable to formulation in a relatively non-toxic vehicle that can be administered to the patient. Urea derivatives have generally low solubility (17), which could be in part mitigated by high potency and slow metabolism, butt formulation is still a challenge. Because **1,728** has such low solubility in water, an organic co-solvent will likely be needed to solubilize it. Thus, the solubility of **1,728** was measured in various solvents commonly used for drug formulation (Table 6) (31). While barely soluble in protic solvents, **1,728** displayed a good solubility (> 20 mg/mL) in numerous organic liquids used in the formulation of drugs such as Carbitol and polyethylene glycol (PEG). As expected, increasing the temperature from 30 to 40°C significantly increased the amount of compound in solution.

**Table 6:** Formulation ease: solubility of **1,728** in various solvents classically used for formulation.

**Temperature**	**30°C**	**40°C**
**Solvent**	**Solubility in mg/mL (mM)**	
DMSO	131 (299)	254 (580)
Ethanol	4 (9)	14 (32)
Propylene glycol	4 (9)	6 (14)
Carbitol	37 (83)	50 (114)
PEG200	85 (195)	91 (207)
PEG300	97 (222)	111 (254)
PEG400	18 (40)	25 (57)
Tween 80	8 (18)	15 (34)
Oleic rich oil	0.13 (0.31)	0.10 (0.22)
Glycerol	0.06 (0.13)	0.18 (0.41)
Phosphate Buffer (0.1 M pH 7.4)	0.005 (0.011)	0.010 (0.022)

## Discussion

From our screen of 2,300 sEH inhibitors, **1,728** is overall the best compound (high potency, good solubility, and stability) for all three species. While less stable, **1,471** is a good alternative for veterinary usage. Although the results obtained herein are only *in vitro*, they suggest that **1,728** will be relatively easy to formulate for dosing in animals. This compound could provide a new approach to relieve pain and inflammation in companion and farm animals through the inhibition of sEH.

Inflammation is necessary for the body to combat and survive infections and damages. However, uncontrolled inflammatory response could lead to cell death, organ damages and contribute to chronic disease such as cancer, heart disease and diabetes. Additionally, both acute and chronic inflammation cause pain as a secondary indication that can reduce quality of life. Acute inflammation causes localized pain that resolves with the injury, while chronic inflammation can result in organ damages and neuropathic pain that is difficult to treat. Inhibition of sEH has been shown to decrease inflammation as well as the inflammatory and neuropathic pain associated with it in animal models (7–10). Recent results show that sEH inhibition reduce pain in horses with chronic laminitis (11–13). The positive effect of the sEH inhibitor used in these studies, **1,728**, was observed either as an additive to the standard-of-care therapy, which was marginally effective (11, 12), or by itself in horses with lipopolysaccharide (LPS)-induced radiocarpal synovitis (13). Upon dosing (0.1–1 mg/kg i.v.), there was a significant and rapid reduction in pain without observed adverse effects (11–13). In a separated study, **1,728** was also able to reduce pain perception in dogs with osteoarthritis (14). Additional promising results have been obtained in an *in vitro* model. Treating chondrocytes, the key cell type that maintain joint function, *in vitro* with EETs, the lipid signaling molecules increased through sEH inhibition *in vivo*, reduced inflammatory markers and was cytoprotective (14). Thus, sEH inhibition, by enhancing concentrations of EETs *in vivo*, could reduce synovial inflammation and subsequent pain leading to reduce synovial inflammation (14). These results indicate that the compound identified here as a potential sEH inhibitor for veterinary medicine application could be efficient *in vivo*.

Beside laminitis in horses and osteoarthritis in dogs, there are many possible therapeutic applications for sEH inhibitors in veterinary medicine. Mirroring human applications (10), along with providing a potential therapy for inflammation and painful diseases in animals, sEH inhibition could also have applications in the treatment of cardiovascular and respiratory diseases. For example, in cattle, the respiratory disease syndrome in feedlots and mastitis of dairy cows commonly due to bacterial infection, are characterized by severe inflammation leading to significant costs to producers in part due to antimicrobial drugs use but also loss of market shares (32). Because epoxy-fatty acids seem to play a role in cattle during mastitis (33), sEH inhibition may provide an alternative therapy for inflammation that may result in improved treatment outcomes and the reduction in the use of antimicrobials in animal agriculture. The selection of a compound, t-TUCB (**1,728**), an sEH inhibitor that that is potent across species and displays good physical properties, will allow researchers and veterinarians to easily study the effect of sEH inhibition in pets and farm animals.

## Conclusion

Put together, **1,728** is overall the best sEH inhibitor (high potency, good solubility and stability) for all the animal species tested herein. This compound through the inhibition of sEH and stabilization of epoxy-fatty acids could provide a new approach to relieve pain and inflammation in companion and farm animals. Toward this end, **1,728** pharmacological properties are currently measured in several animal species toward testing it in animal clinical trials soon.

## Data Availability Statement

All datasets generated for this study are included in the article/Supplementary Material.

## Author Contributions

VM, BH, SK, S-HH, and AG: provide key materials. DS, TH, SK, KL, and CM: run experiments and acquire the data. TH, KL, and CM: data analysis. DS, BDH, and CM: study design. TH, SK, S-HH, KL, VM, AG, BDH, and CM: manuscript writing and editing. All authors contributed to the article and approved the submitted version.

## Conflict of Interest

The University of California holds patents on the sEH inhibitors used in this study as well as their use to treat inflammation, inflammatory pain, and neuropathic pain. AG, BDH, and CM are inventors on some of these patents. BDH is cofounder of EicOsis L.L.C., a startup company advancing sEH inhibitors as potential therapeutics. The remaining authors declare that the research was conducted in the absence of any commercial or financial relationships that could be construed as a potential conflict of interest.
