# Supplementary material for: Selection of Potent Inhibitors of Soluble Epoxide Hydrolase for Usage in Veterinary Medicine
Source: Front Vet Sci. 2020 Aug 26;7:580. doi: 10.3389/fvets.2020.00580 (PMC7479175; doi:10.3389/fvets.2020.00580)
Supplement: Supplementary file 1 [file Data_Sheet_1.PDF]

Selection of potent inhibitors of soluble epoxide hydrolase for usage in veterinary medicine

Supplemental Data

Diyala S.Shihadih,[a] Todd R. Harris,[a] Sean D. Kodani,[a] Sung-Hee Hwang,[a] Kin Sing Stephen Lee,[a,b] Vengai Mavangira,[c] Briana Hamamoto,[d] Alonso Guedes,[d,e] Bruce D. Hammock[a], and Christophe Morisseau[a,*]

[a] Department of Entomology and Nematology, and U.C. Davis Comprehensive Cancer Center, University of California Davis, Davis, CA 95616 USA
[b] Department of Pharmacology and Toxicology, and Department of Chemistry, Michigan State University, East Lansing, MI 48824 USA
[c] Department of Large Animal Clinical Sciences, College of Veterinary Medicine, Michigan State University, East Lansing 48824 USA
[d] Department of Surgical and Radiological Sciences, School of Veterinary Medicine, University of California, Davis, CA, USA
[e] Department of Veterinary Clinical Sciences, College of Veterinary Medicine, University of Minnesota, St Paul, MN, USA

* Corresponding author. Tel.: +1-530-752-6571.
E-mail address: chmorisseau@ucdavis.edu (C. Morisseau).

Table S1: Result of primary screening with [I] = 100 nM, from the primary (P.) end-point screening and the secondary (S.) kinetic mode screening.

Plt. #	Row #	Col. #	EHI #	Structure	Horse sEH Inhibition (%)		Cat sEH Inhibition (%)		Dog sEH Inhibition (%)	
					P.	S.	P.	S.	P.	S.
**1**	**A**	**12**	1132	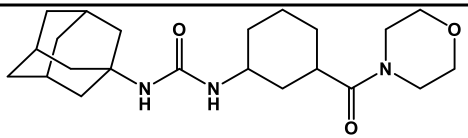	98	85	< 90	79	< 90	32
**1**	**B**	**5**	1077	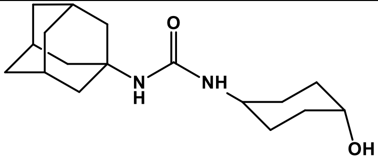	< 90	62	< 90	29	99	29
**1**	**C**	**5**	1078	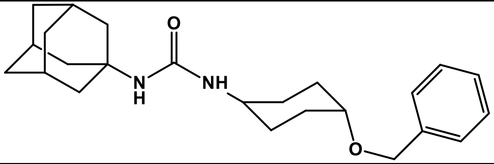	100	85	96	103	90	84
**1**	**D**	**12**	1135	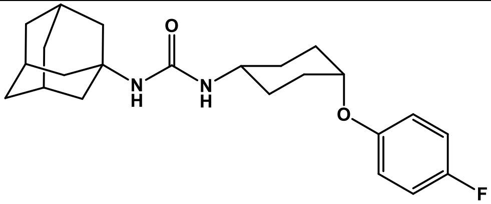	111	80	91	77	96	58
**1**	**E**	**7**	1096	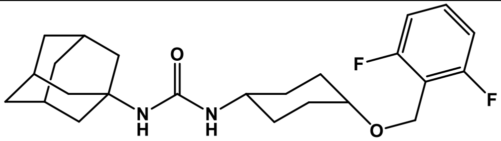	102	76	102	104	106	73
**1**	**E**	**11**	1128	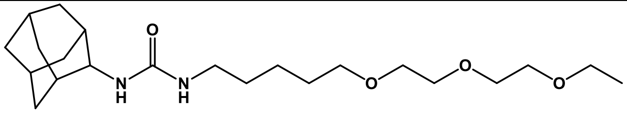	110	92	95	88	90	64
**1**	**F**	**10**	1121	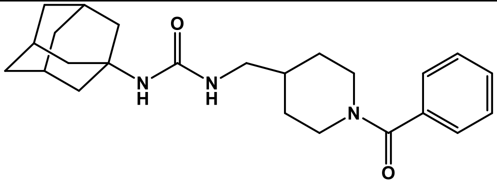	114	84	< 90	94	< 90	56

**1**	**F**	**11**	1129	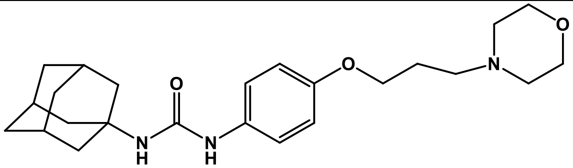	116	82	< 90	94	< 90	7
**1**	**F**	**12**	1137	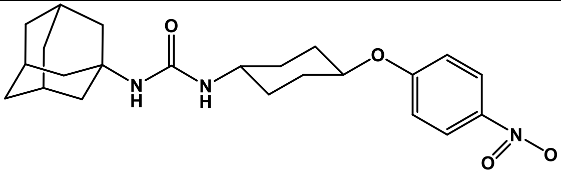	118	76	99	89	< 90	65
**1**	**G**	**4**	1074	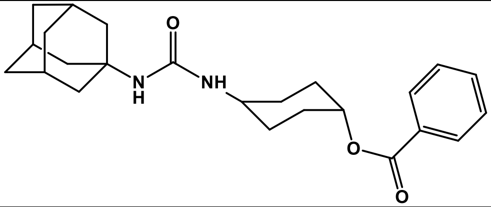	91	84	< 90	71	91	82
**1**	**G**	**7**	1098	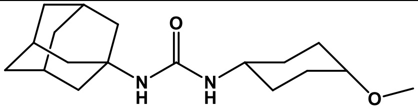	93	84	< 90	80	90	50
**1**	**G**	**8**	1106	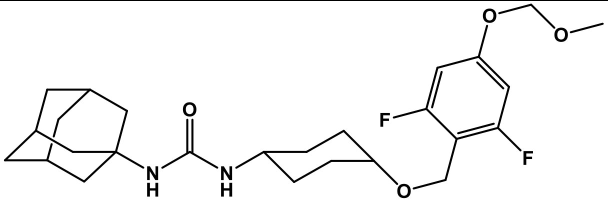	< 90	83	108	104	< 90	71
**1**	**G**	**11**	1130	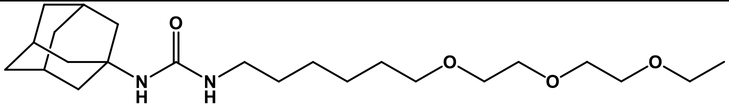	118	89	91	94	< 90	51
**1**	**G**	**12**	1138	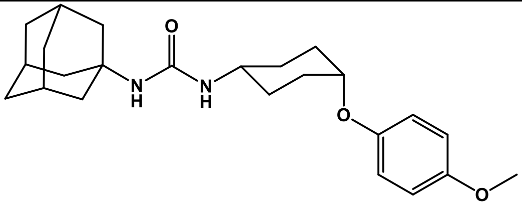	112	75	< 90	62	< 90	63
**1**	**H**	**3**	1067	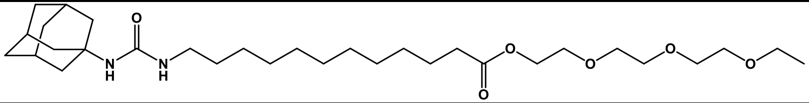	94	61	100	75	< 90	49

**1**	**H**	**7**	1099	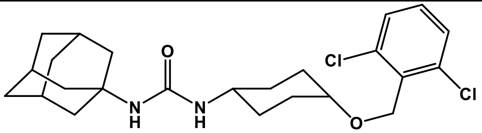	< 90	68	< 90	34	90	-14
**1**	**H**	**11**	1131	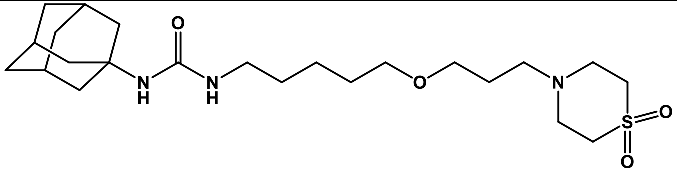	114	89	< 90	69	< 90	30
**2**	**A**	**8**	1186	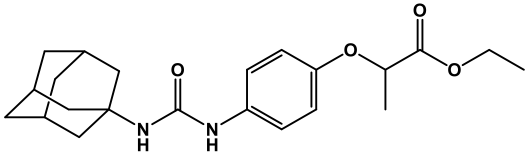	< 90	80	94	104	< 90	63
**2**	**A**	**10**	1202	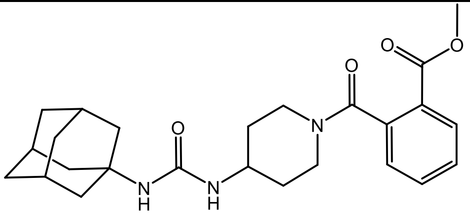	103	85	102	93	< 90	72
**2**	**A**	**11**	1210	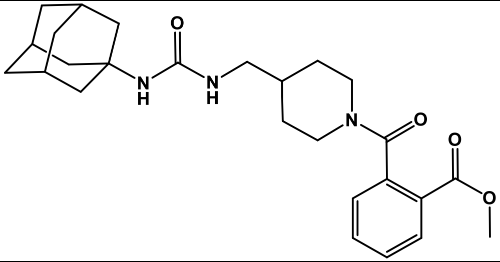	101	91	101	94	< 90	75
**2**	**B**	**3**	1147	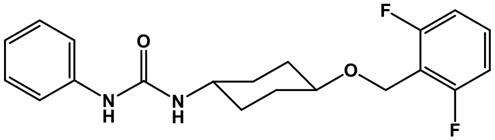	94	92	93	92	< 90	40
**2**	**B**	**5**	1163	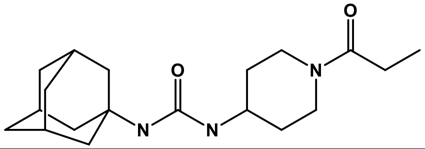	97	83	92	61	< 90	48

**2**	**B**	**10**	1203	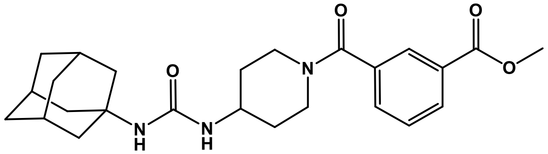	< 90	91	101	103	< 90	81
**2**	**B**	**11**	1211	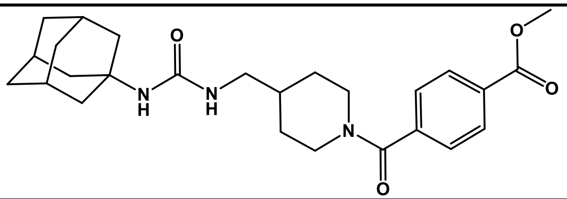	< 90	65	91	38	< 90	15
**2**	**C**	**2**	1140	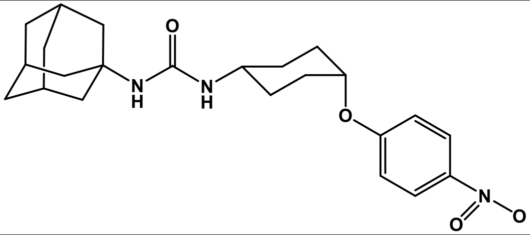	90	76	100	91	100	81
**2**	**C**	**9**	1196	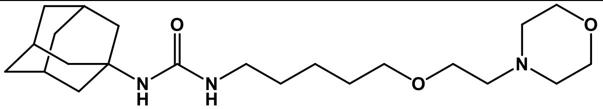	97	82	90	91	< 90	45
**2**	**C**	**10**	1204	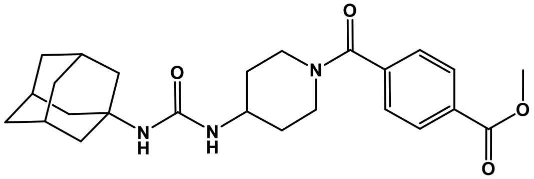	109	75	102	99	< 90	72
**2**	**D**	**2**	1141	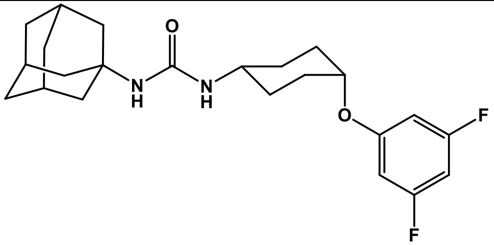	97	88	99	102	99	80
**2**	**D**	**9**	1197	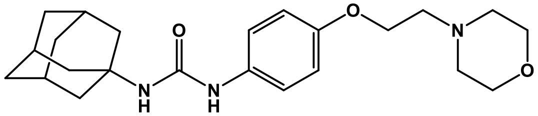	92	89	99	97	99	47

**2**	**E**	**6**	1174	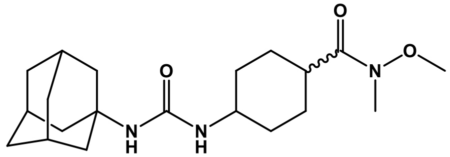	103	84	100	95	< 90	71
**2**	**E**	**9**	1198	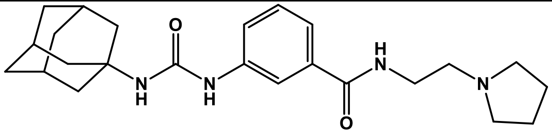	< 90	84	95	77	< 90	28
**2**	**E**	**10**	1206	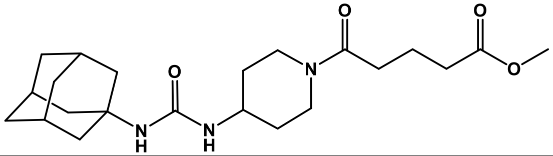	< 90	84	93	92	< 90	59
**2**	**F**	**4**	1159	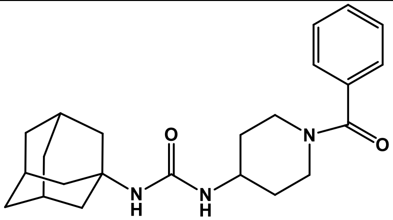	102	85	93	109	< 90	76
**2**	**F**	**5**	1167	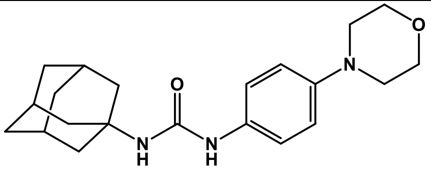	98	84	< 90	96	< 90	46
**2**	**F**	**7**	1183	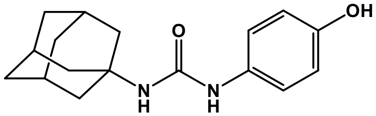	90	80	< 90	25	< 90	24
**2**	**F**	**8**	1191	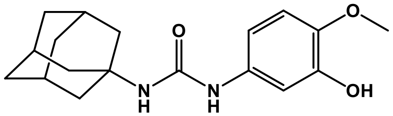	90	83	< 90	50	< 90	25
**2**	**F**	**10**	1207	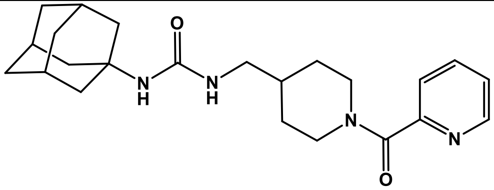	111	89	92	91	< 90	54

**2**	**F**	**11**	1215	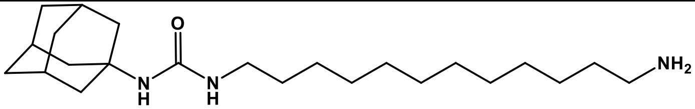	104	88	103	96	< 90	69
**2**	**G**	**7**	1184	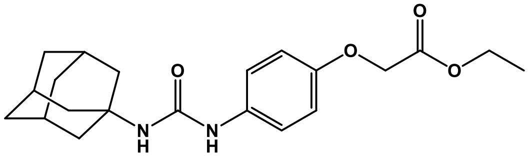	102	90	93	103	< 90	66
**2**	**G**	**10**	1208	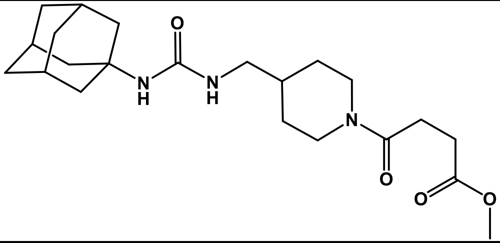	102	75	< 90	78	< 90	23
**2**	**G**	**11**	1217	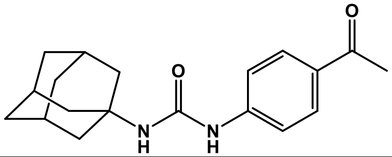	99	79	< 90	52	< 90	25
**2**	**H**	**9**	1201	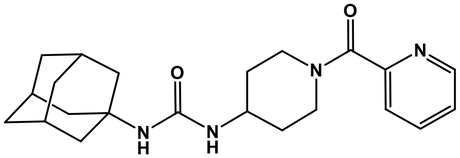	97	73	< 90	85	< 90	41
**3**	**A**	**5**	1450	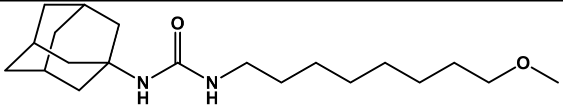	90	87	93	101	< 90	75
**3**	**A**	**10**	1517	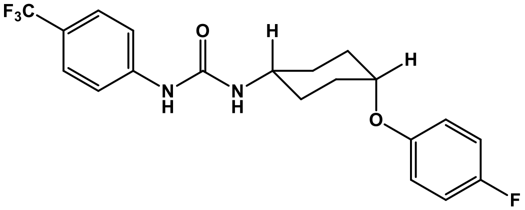	102	91	< 90	94	< 90	55
**3**	**B**	**3**	1435	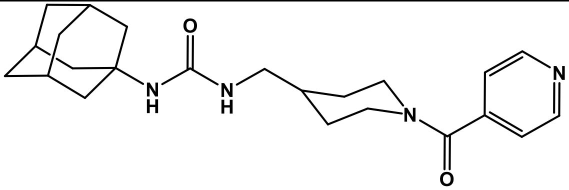	< 90	17	97	0	< 90	5

**3**	**C**	**10**	1519	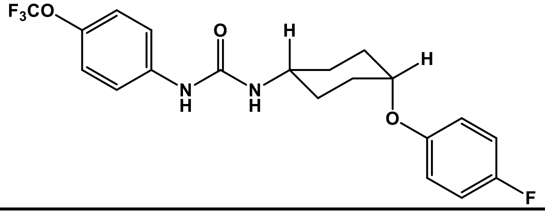	97	94	93	95	93	46
**3**	**C**	**11**	1551	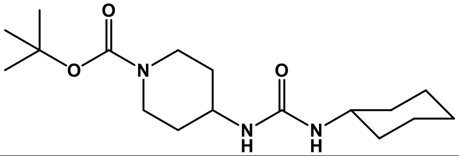	92	84	< 90	90	< 90	52
**3**	**D**	**7**	1469	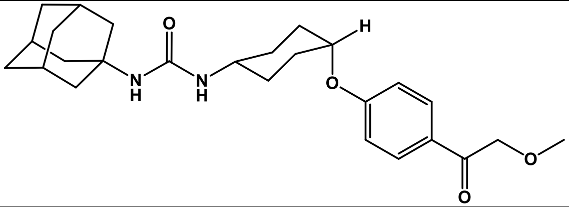	< 90	67	< 90	65	90	30
**3**	**D**	**8**	1504	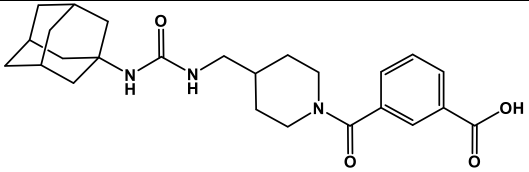	< 90	80	92	95	93	63
**3**	**D**	**10**	1520	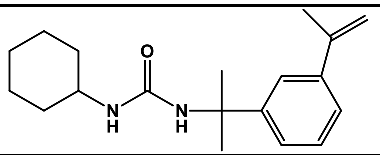	108	93	97	98	< 90	69
**3**	**E**	**7**	1470	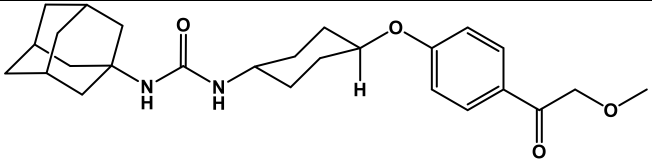	< 90	83	< 90	82	100	69
**3**	**F**	**3**	1439	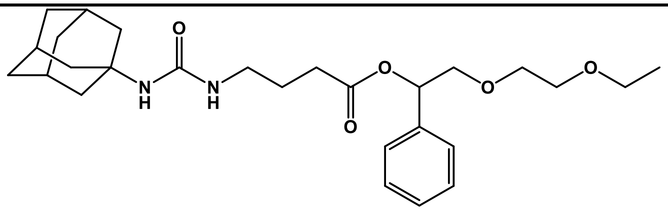	93	89	94	94	< 90	55

**3**	**F**	**4**	1447	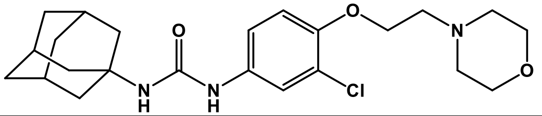	93	89	90	103	< 90	73
**3**	**F**	**7**	1471	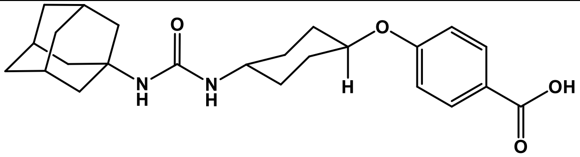	103	89	102	113	94	89
**3**	**F**	**9**	1514	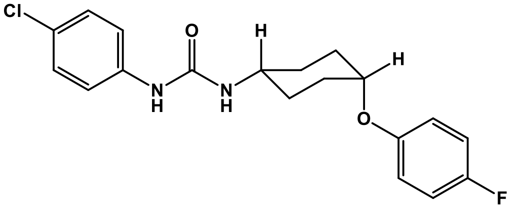	108	78	100	98	< 90	48
**3**	**F**	**10**	1522	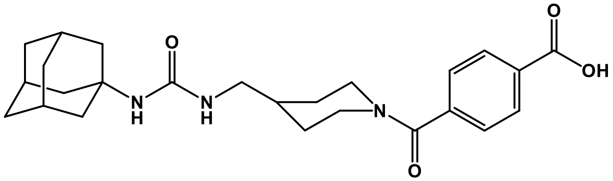	108	80	103	102	< 90	57
**3**	**G**	**9**	1515	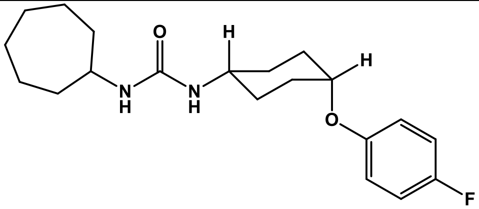	114	87	104	108	90	85
**3**	**G**	**10**	1523	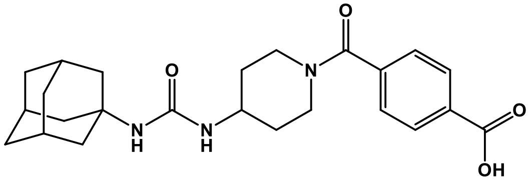	111	83	99	93	< 90	68
**3**	**G**	**11**	1556	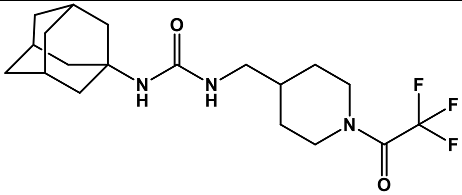	107	84	99	92	< 90	65

**3**	**H**	**4**	1449	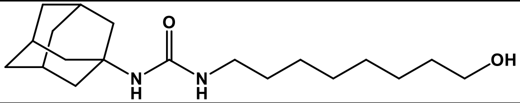	< 90	88	97	100	< 90	64
**3**	**H**	**7**	1500	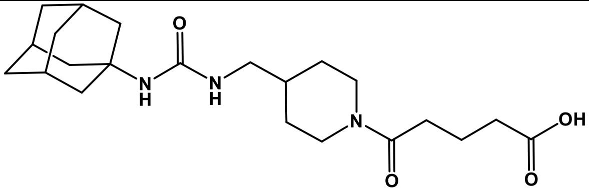	< 90	79	93	86	< 90	46
**3**	**H**	**9**	1516	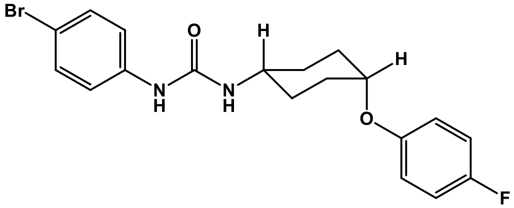	91	82	< 90	94	< 90	22
**3**	**H**	**12**	1565	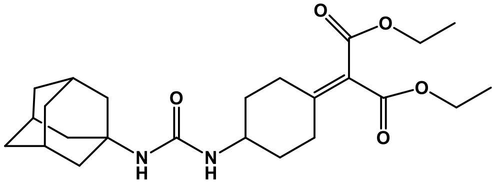	115	88	103	94	88	82
**4**	**A**	**3**	1241	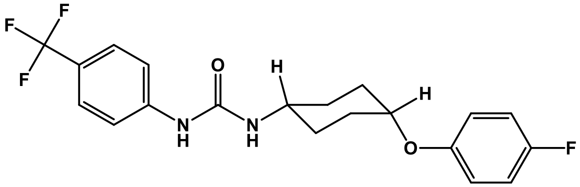	94	82	90	91	< 90	35
**4**	**A**	**4**	1249	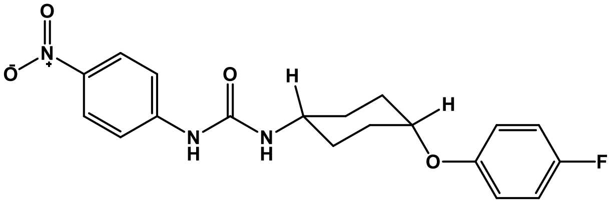	< 90	84	93	99	< 90	57
**4**	**B**	**2**	1234	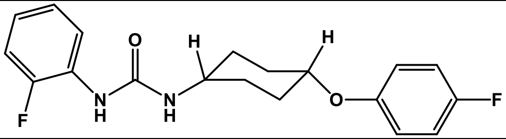	91	88	98	106	< 90	68

**4**	**B**	**3**	1242	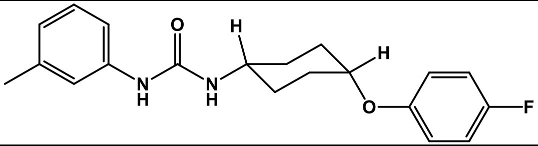	94	87	95	107	86	81
**4**	**B**	**7**	1274	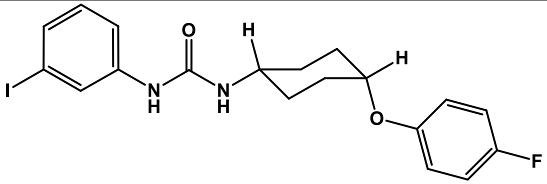	< 90	84	95	89	< 90	65
**4**	**C**	**2**	1235	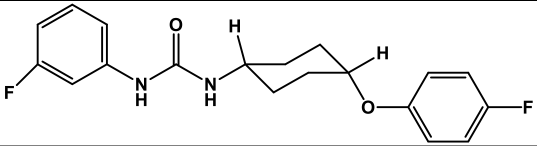	93	90	99	106	< 90	69
**4**	**C**	**5**	1259	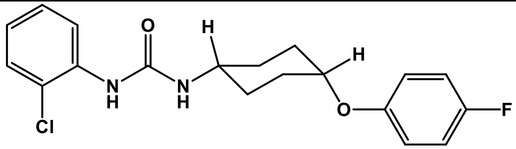	< 90	81	93	101	< 90	62
**4**	**C**	**12**	1318	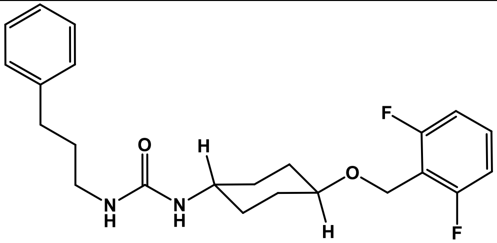	103	93	98	110	< 90	80
**4**	**D**	**3**	1244	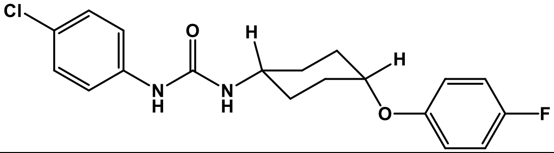	90	84	96	100	< 90	21
**4**	**E**	**2**	1237	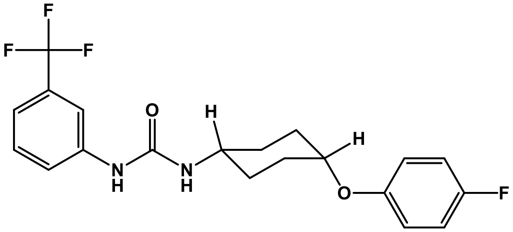	97	80	< 90	88	< 90	68

**4**	**E**	**4**	1253	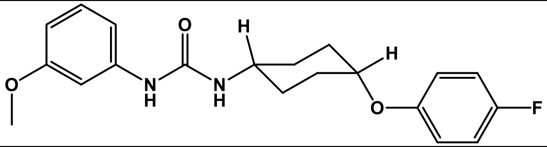	94	87	98	98	< 90	45
**4**	**E**	**5**	1261	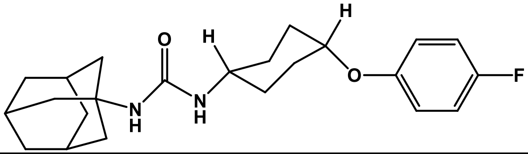	< 90	87	97	102	< 90	75
**4**	**E**	**6**	1269	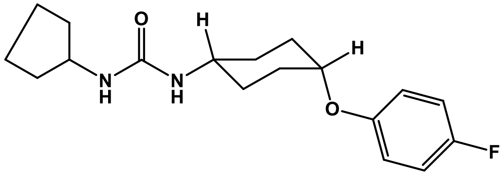	< 90	87	100	100	< 90	57
**4**	**E**	**7**	1277	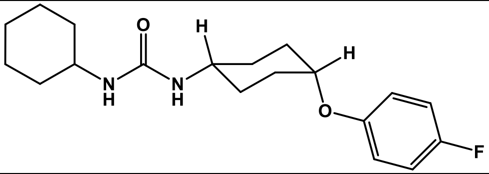	92	92	97	104	< 90	78
**4**	**E**	**11**	1311	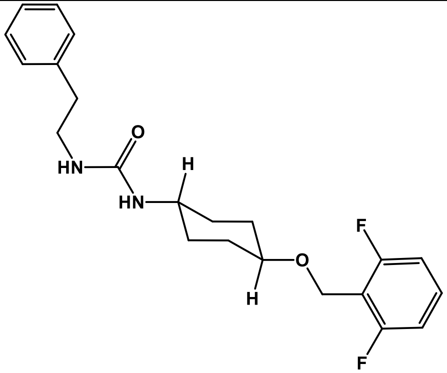	101	91	93	103	< 90	62
**4**	**F**	**4**	1254	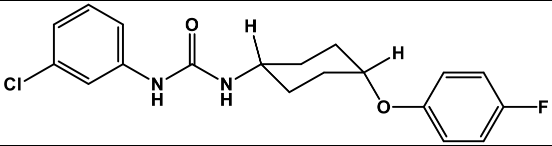	93	90	96	100	97	81

**4**	**F**	**6**	1270	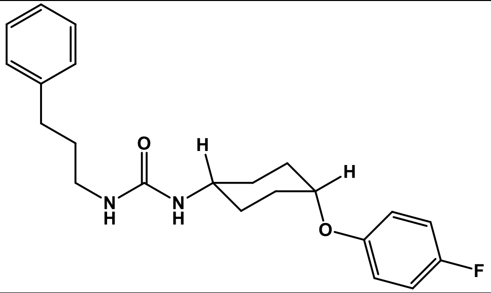	99	92	102	104	< 90	82
**4**	**F**	**7**	1278	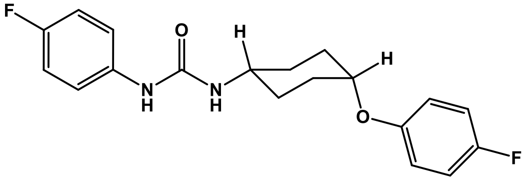	91	83	95	95	< 90	68
**4**	**F**	**8**	1287	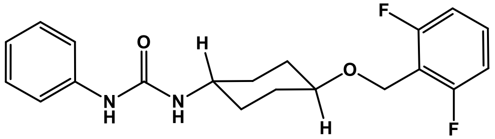	91	89	< 90	103	< 90	52
**4**	**G**	**2**	1239	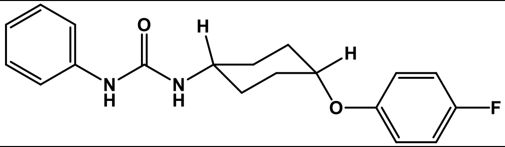	97	86	97	99	< 90	63
**4**	**G**	**5**	1263	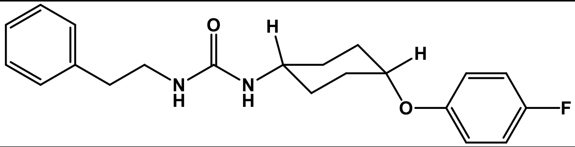	102	86	96	88	< 90	53
**4**	**G**	**6**	1271	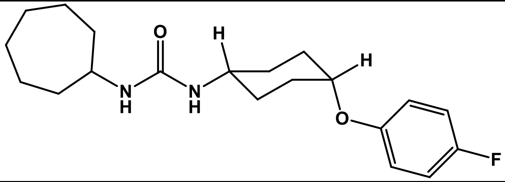	96	88	104	120	< 90	87
**4**	**G**	**7**	1279	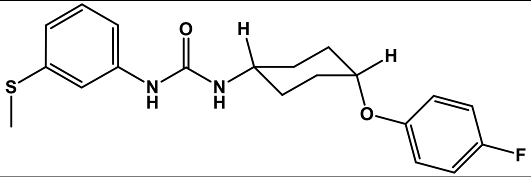	91	85	< 90	91	< 90	44

**4**	**G**	**8**	1288	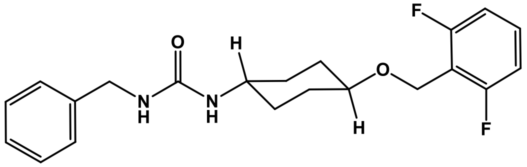	97	85	< 90	93	< 90	64
**4**	**G**	**9**	1296	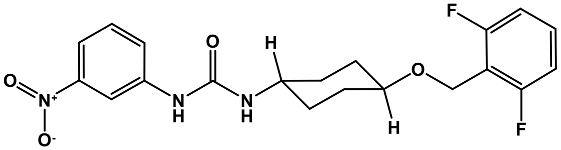	92	82	< 90	87	< 90	62
**4**	**G**	**10**	1304	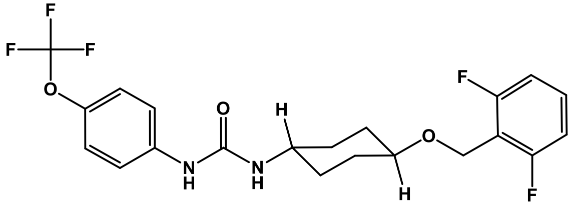	92	81	< 90	78	< 90	32
**4**	**H**	**2**	1240	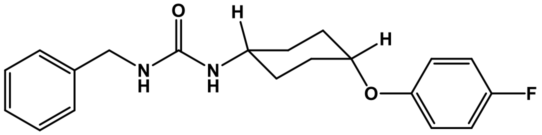	97	86	< 90	83	< 90	66
**4**	**H**	**3**	1248	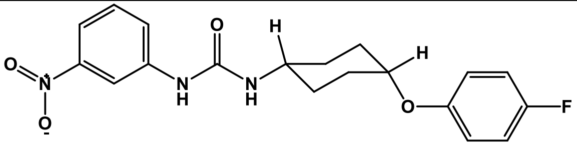	113	87	95	91	< 90	67
**4**	**H**	**4**	1256	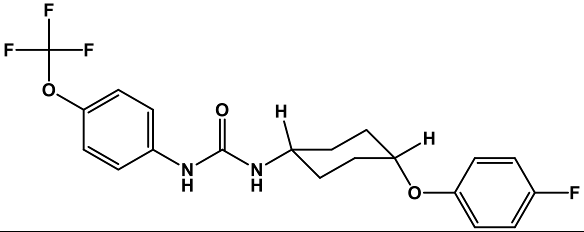	98	88	93	88	< 90	46
**4**	**H**	**6**	1272	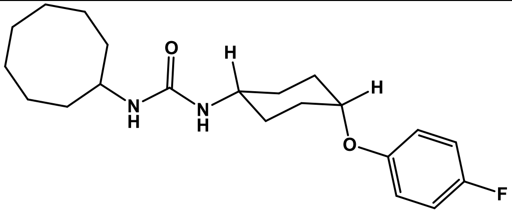	120	88	102	101	93	82

**4**	**H**	**9**	1297	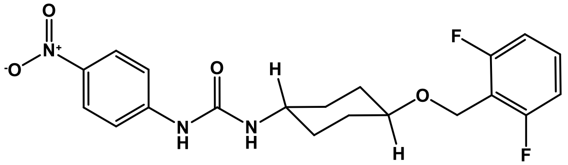	102	80	< 90	74	< 90	14
**4**	**H**	**11**	1314	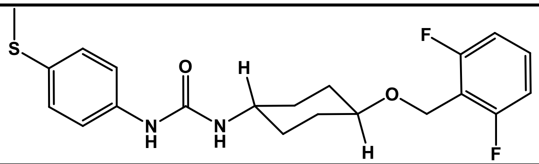	91	74	< 90	63	< 90	19
**4**	**H**	**12**	1327	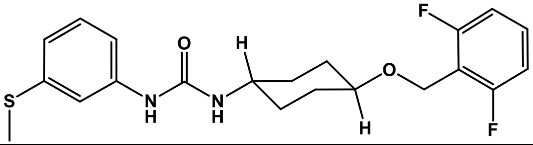	96	79	< 90	77	< 90	20
**6**	**A**	**6**	1619	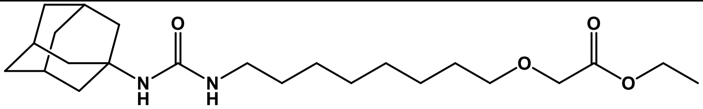	95	81	97	94	< 90	64
**6**	**A**	**10**	1666	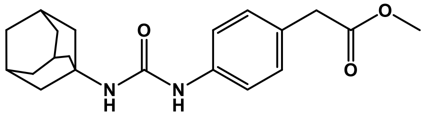	102	88	101	101	91	52
**6**	**B**	**5**	1612	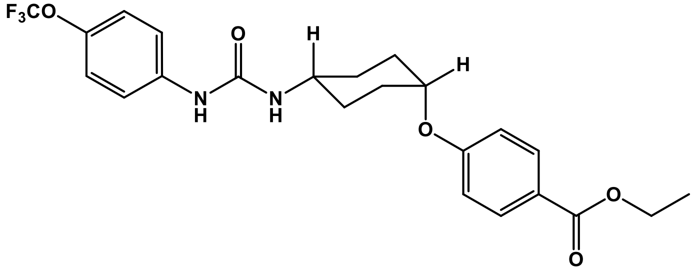	< 90	73	100	59	< 90	18
**6**	**B**	**6**	1620	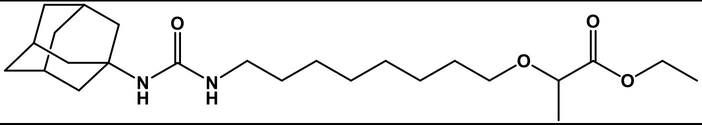	98	83	112	96	90	85
**6**	**B**	**11**	1675	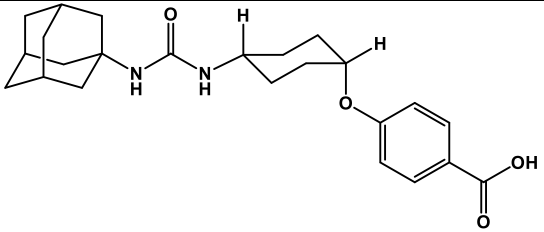	99	89	109	99	97	85

**6**	**C**	**3**	1590	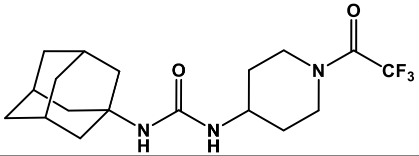	92	86	< 90	88	< 90	76
**6**	**C**	**4**	1605	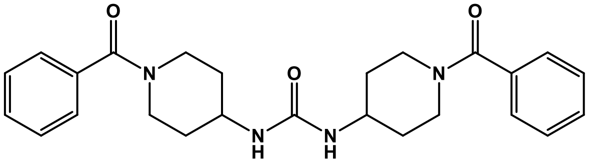	< 90	83	96	87	< 90	61
**6**	**C**	**6**	1621	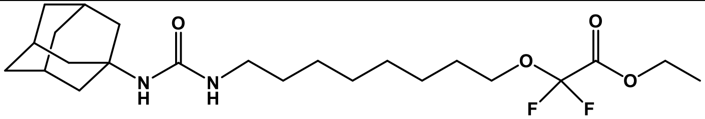	94	85	97	100	90	80
**6**	**C**	**9**	1660	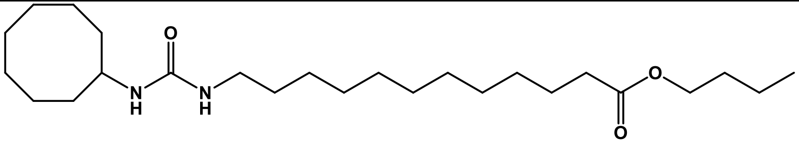	< 90	75	104	46	108	38
**6**	**C**	**10**	1668	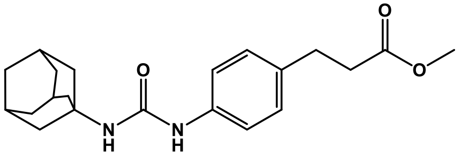	91	85	98	99	< 90	71
**6**	**C**	**12**	1684	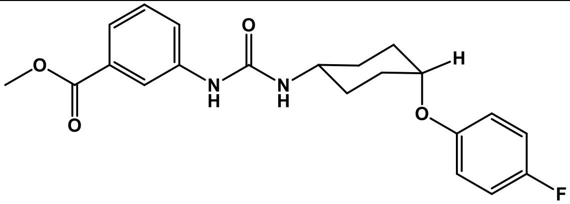	96	84	105	92	< 90	68
**6**	**D**	**3**	1591	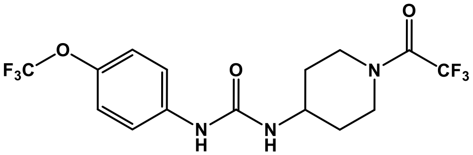	102	88	< 90	40	< 90	1
**6**	**D**	**4**	1606	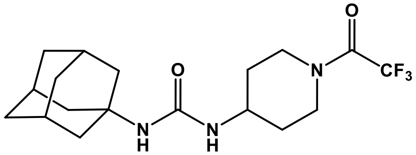	91	85	< 90	87	< 90	79

**6**	**D**	**6**	1622	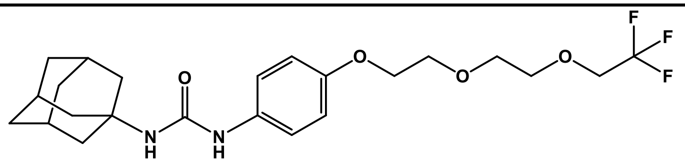	104	85	108	96	108	73
**6**	**E**	**2**	1566	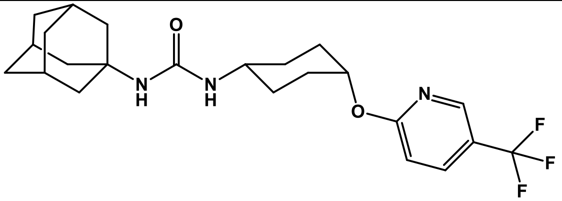	90	84	< 90	81	< 90	74
**6**	**E**	**5**	1615	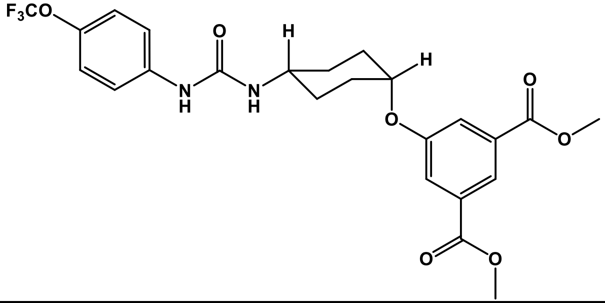	93	77	< 90	18	< 90	23
**6**	**E**	**9**	1662	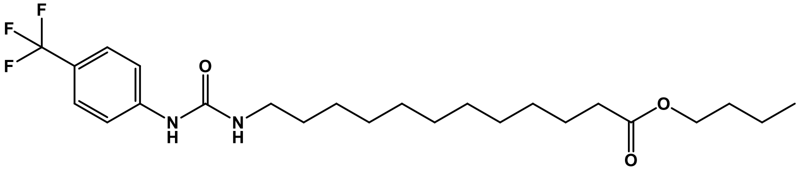	< 90	19	92	3	< 90	6
**6**	**E**	**12**	1686	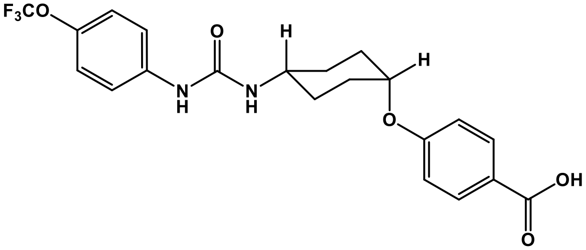	107	88	119	96	< 90	80
**6**	**F**	**4**	1608	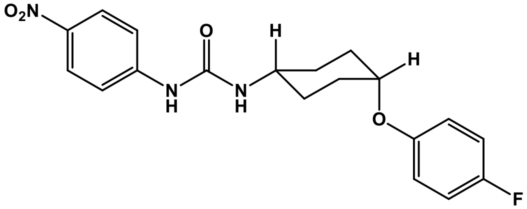	103	86	106	94	< 90	67

**6**	**F**	**5**	1616	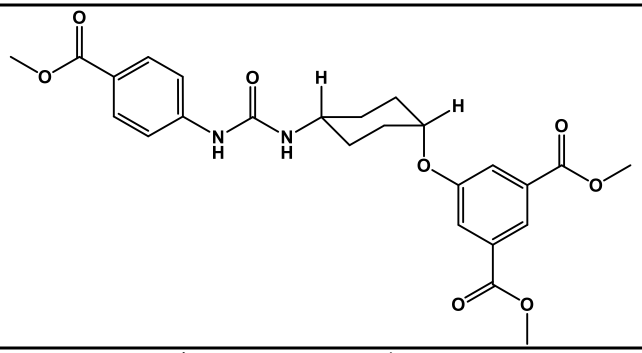	101	85	< 90	41	< 90	20
**6**	**F**	**7**	1647	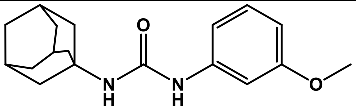	100	87	99	89	< 90	68
**6**	**F**	**9**	1663	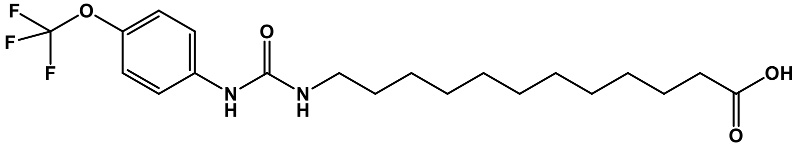	< 90	85	93	102	< 90	86
**6**	**F**	**10**	1671	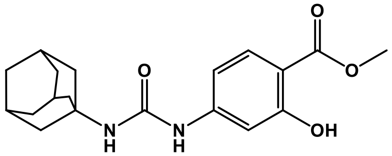	103	77	96	68	< 90	49
**6**	**F**	**11**	1679	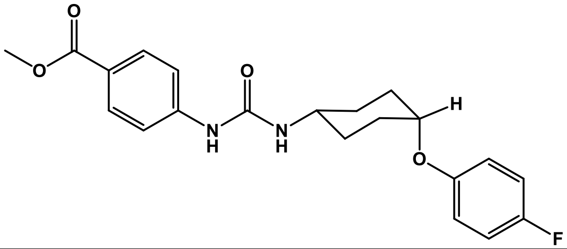	106	88	< 90	92	< 90	58
**6**	**G**	**5**	1617	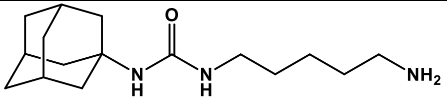	104	64	< 90	21	< 90	32
**6**	**G**	**7**	1648	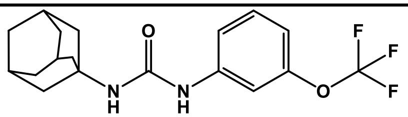	111	87	103	96	< 90	51
**6**	**G**	**12**	1688	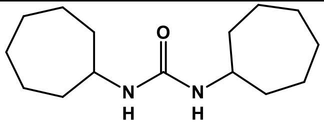	100	86	91	97	< 90	68

**6**	**H**	**3**	1602	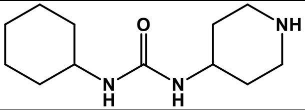	94	72	< 90	34	< 90	24
**6**	**H**	**4**	1610	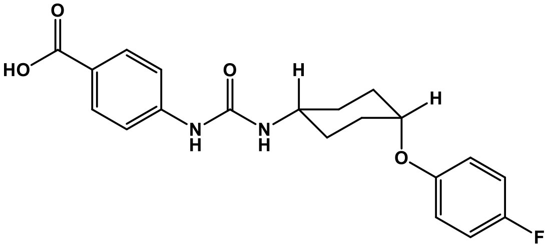	106	83	91	88	< 90	36
**6**	**H**	**5**	1618	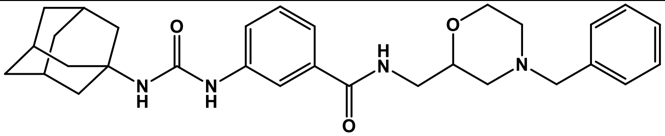	94	82	< 90	48	< 90	26
**6**	**H**	**7**	1649	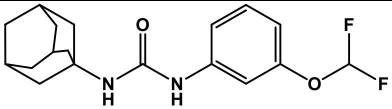	112	89	101	88	< 90	65
**6**	**H**	**8**	1657	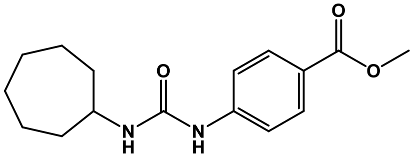	102	86	< 90	79	< 90	34
**7**	**A**	**8**	140	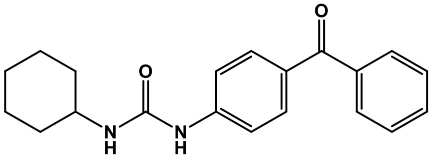	93	84	< 90	96	< 90	40
**7**	**B**	**4**	1707	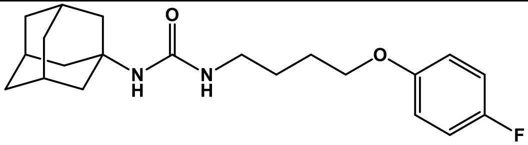	101	87	94	99	< 90	83
**7**	**B**	**9**	359	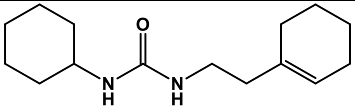	103	88	< 90	93	< 90	30
**7**	**B**	**10**	437	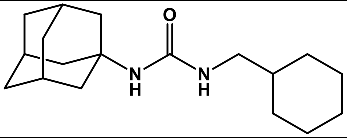	101	87	< 90	87	< 90	69

**7**	**B**	**12**	578	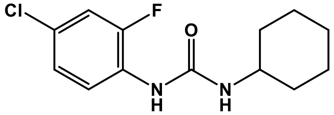	90	80	< 90	77	< 90	34
**7**	**C**	**8**	179	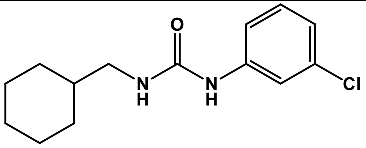	93	85	< 90	93	< 90	75
**7**	**C**	**9**	360	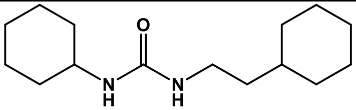	103	89	95	99	< 90	75
**7**	**C**	**10**	438	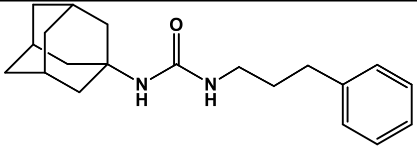	104	88	92	99	90	82
**7**	**D**	**3**	1701	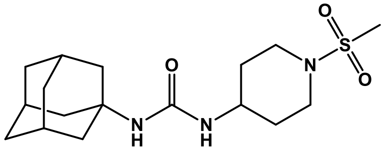	106	86	< 90	76	< 90	55
**7**	**D**	**7**	22	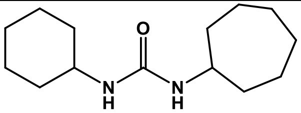	106	86	95	88	< 90	59
**7**	**D**	**8**	180	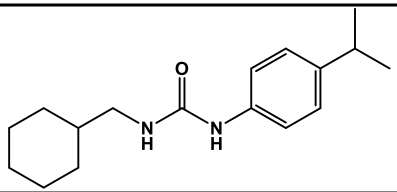	107	89	92	106	< 90	79
**7**	**D**	**10**	473	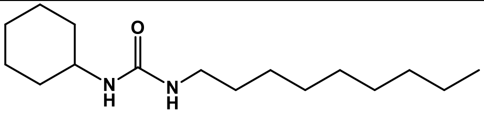	98	83	< 90	101	< 90	62
**7**	**E**	**3**	1702	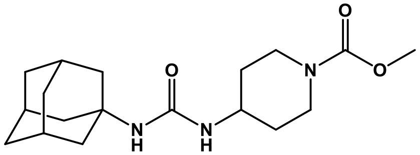	101	85	< 90	77	< 90	63

**7**	**E**	**8**	192	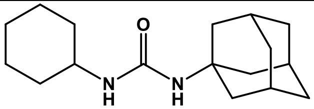	102	87	< 90	108	< 90	85
**7**	**E**	**9**	412	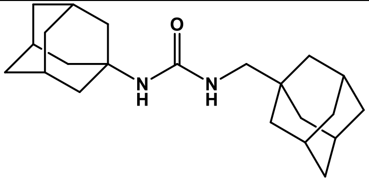	94	86	< 90	68	< 90	77
**7**	**E**	**10**	508	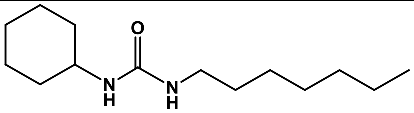	105	90	< 90	90	< 90	76
**7**	**F**	**4**	1711	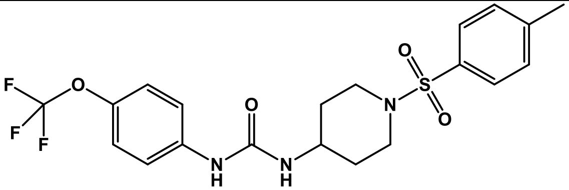	108	85	< 90	58	< 90	18
**7**	**F**	**7**	66	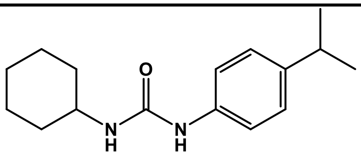	105	86	< 90	99	< 90	22
**7**	**F**	**8**	257	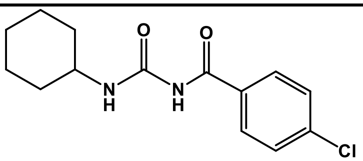	91	83	< 90	75	< 90	30
**7**	**G**	**6**	1728	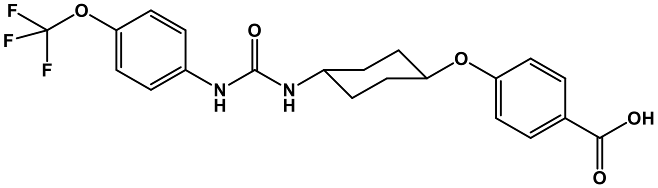	107	89	95	100	90	81
**7**	**G**	**7**	118	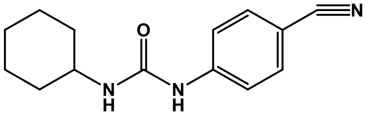	108	85	< 90	98	< 90	42

**7**	**G**	**8**	262	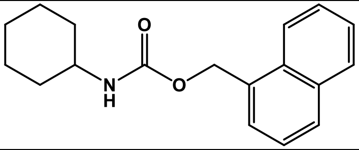	91	87	< 90	82	< 90	48
**7**	**G**	**9**	428	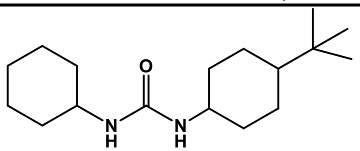	106	85	107	98	< 90	74
**7**	**G**	**10**	538	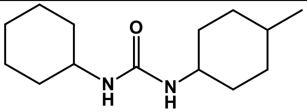	110	79	91	63	< 90	40
**7**	**H**	**3**	1705	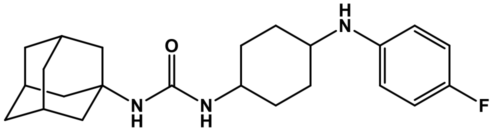	98	78	< 90	70	90	71
**7**	**H**	**4**	1713	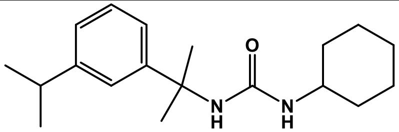	96	87	< 90	70	< 90	60
**7**	**H**	**8**	343	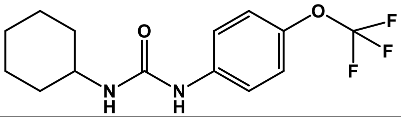	97	84	< 90	78	< 90	9
**7**	**H**	**9**	434	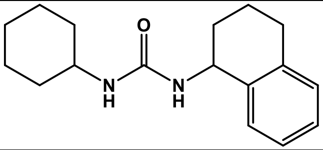	105	88	91	100	< 90	73
**8**	**B**	**7**	38	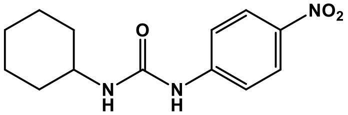	100	79	< 90	51	< 90	12
**8**	**B**	**12**	108	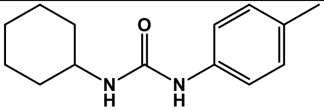	97	82	< 90	90	< 90	15
**8**	**C**	**10**	75	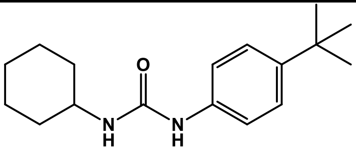	95	86	< 90	98	< 90	34

**8**	**E**	**3**	616	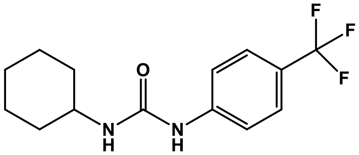	94	79	< 90	53	< 90	24
**8**	**E**	**8**	57	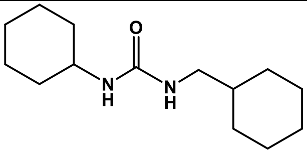	101	88	102	90	91	78
**8**	**F**	**3**	629	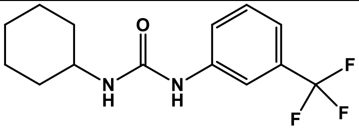	103	90	< 90	64	< 90	47
**8**	**F**	**8**	58	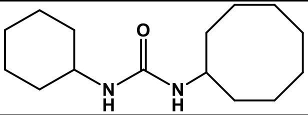	103	92	97	101	< 90	75
**8**	**G**	**7**	44	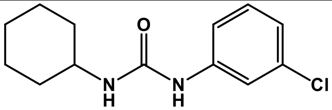	97	92	< 90	66	< 90	50
**8**	**G**	**11**	104	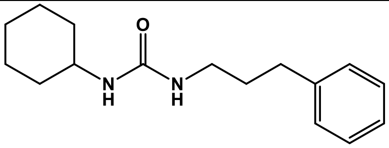	106	91	< 90	95	< 90	53
**8**	**H**	**12**	117	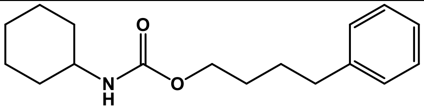	103	41	< 90	31	< 90	4
**9**	**A**	**4**	156	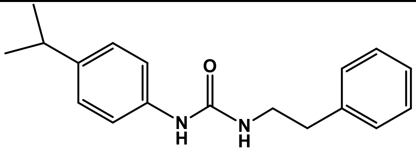	101	94	< 90	70	< 90	16
**9**	**A**	**11**	262	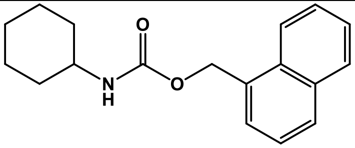	96	81	< 90	73	< 90	36

**9**	**B**	**2**	124	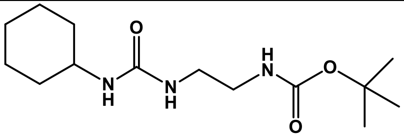	99	85	< 90	91	< 90	60
**9**	**C**	**6**	175	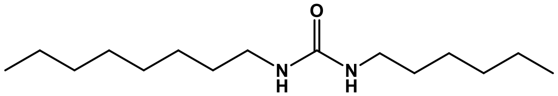	95	81	< 90	67	< 90	40
**9**	**D**	**2**	126	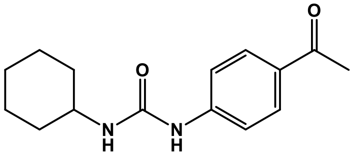	99	91	< 90	95	< 90	70
**9**	**D**	**5**	168	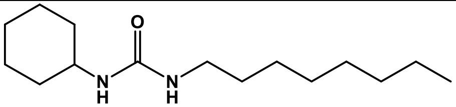	104	88	95	101	< 90	78
**9**	**D**	**10**	253	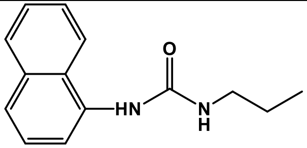	100	87	< 90	90	< 90	35
**9**	**E**	**5**	169	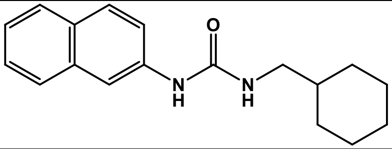	93	89	< 90	84	< 90	51
**9**	**E**	**7**	189	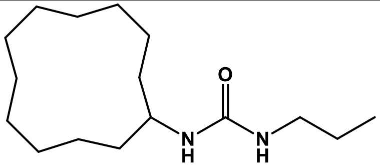	< 90	56	96	76	< 90	21
**9**	**F**	**5**	170	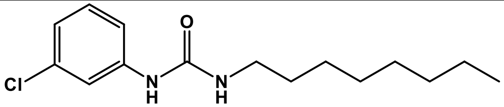	101	83	< 90	80	< 90	27
**9**	**F**	**6**	178	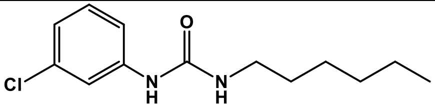	104	84	< 90	68	< 90	46
**9**	**G**	**6**	181	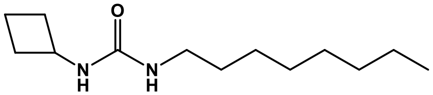	107	89	98	95	90	60

**9**	**G**	**10**	257	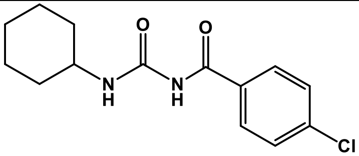	108	86	< 90	90	< 90	55
**9**	**G**	**12**	297	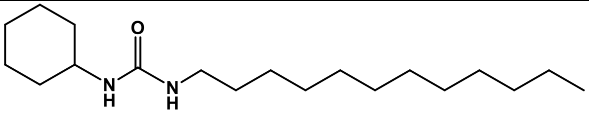	91	55	< 90	18	< 90	17
**9**	**H**	**2**	143	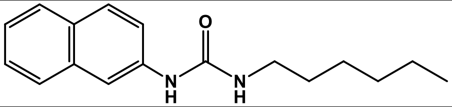	93	85	< 90	63	< 90	13
**9**	**H**	**10**	261	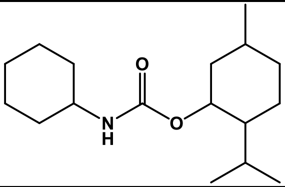	107	93	113	88	< 90	68
**10**	**A**	**11**	438	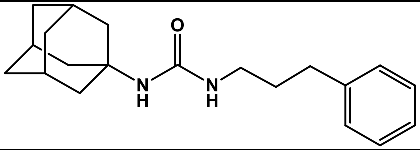	104	92	102	103	< 90	77
**10**	**B**	**5**	359	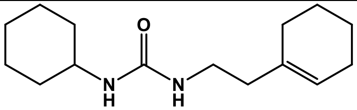	100	91		92	< 90	21
**10**	**C**	**5**	360	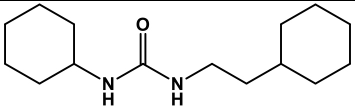	102	86	100	94	< 90	80
**10**	**D**	**5**	361	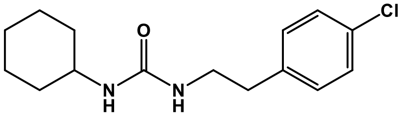	97	86	98	56	< 90	11
**10**	**D**	**11**	442	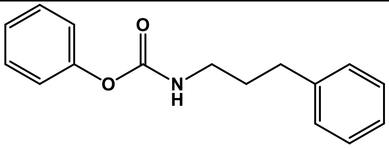	105	88		74	< 90	74
**10**	**E**	**10**	434	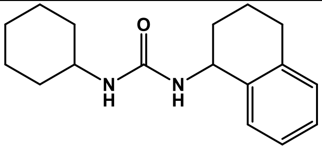	103	90	99	93	< 90	78

**10**	**F**	**10**	435	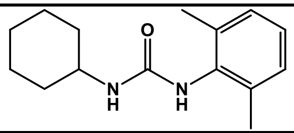	95	73	93	59	< 90	27
**10**	**G**	**9**	428	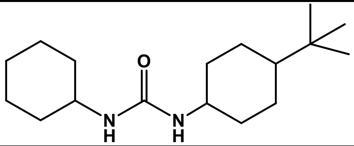	105	92	105	95	< 90	76
**10**	**H**	**10**	437	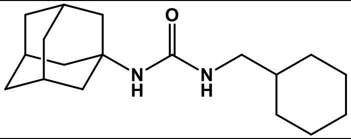	92	91	< 90	86	< 90	76
**11**	**G**	**5**	544	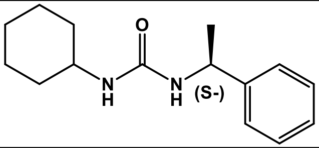	100	87	< 90	75	< 90	39
**11**	**H**	**5**	545	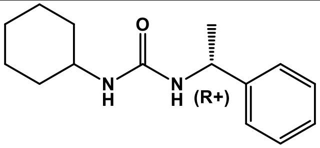	92	24	< 90	28	< 90	14
**11**	**H**	**7**	562	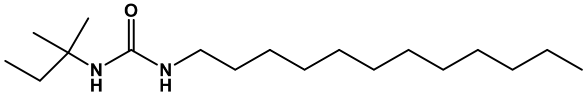	96	15	< 90	27	< 90	-1
**11**	**H**	**10**	622	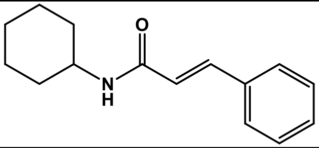	93	30	< 90	28	< 90	26
**11**	**H**	**11**	633	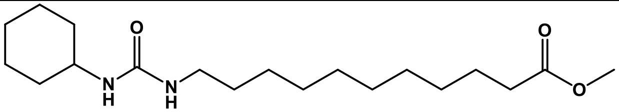	105	89	< 90	98	< 90	83
**11**	**H**	**12**	641	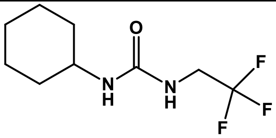	104	63	< 90	56	< 90	26
**12**	**A**	**12**	783	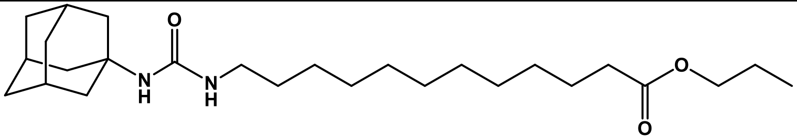	< 90	58	97	27	< 90	27

**12**	**B**	**3**	651	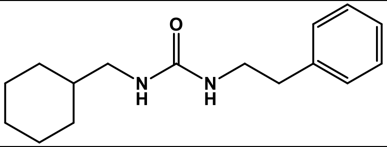	98	93	< 90	65	< 90	37
**12**	**B**	**5**	705	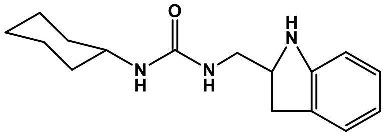	98	85	< 90	79	< 90	21
**12**	**B**	**12**	784	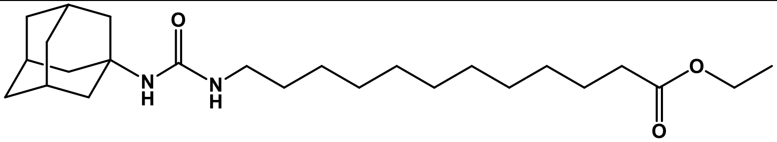	< 90	70	101	48	90	54
**12**	**C**	**12**	786	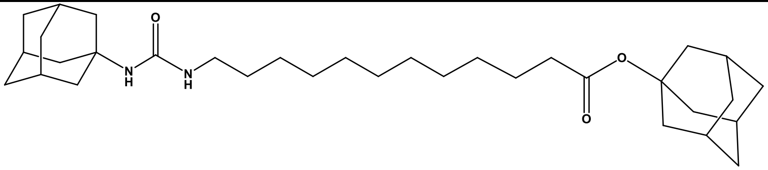	< 90	29	< 90	33	90	6
**12**	**D**	**5**	707	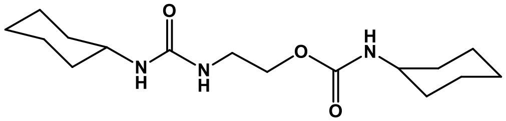	102	94	< 90	87	< 90	58
**12**	**D**	**12**	787	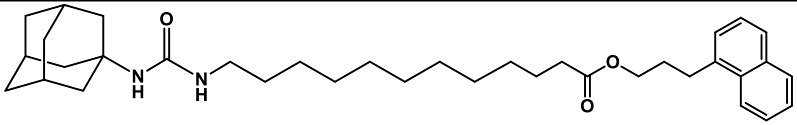	< 90	37	97	30	90	64
**12**	**E**	**4**	700	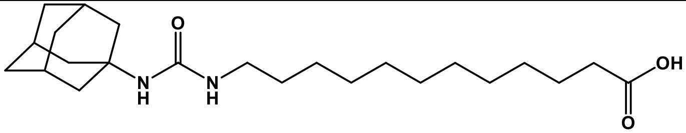	94	85	94	99	92	80
**12**	**F**	**11**	780	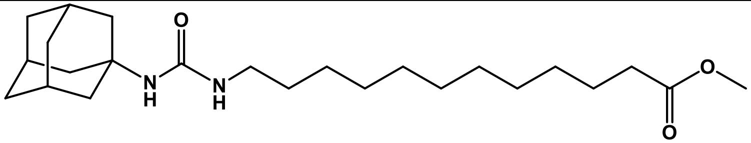	< 90	73	96	75	90	73
**12**	**F**	**12**	789	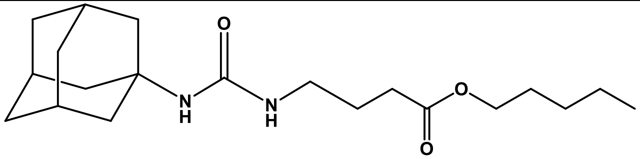	98	87	98	100	< 90	80

**12**	**G**	**11**	781	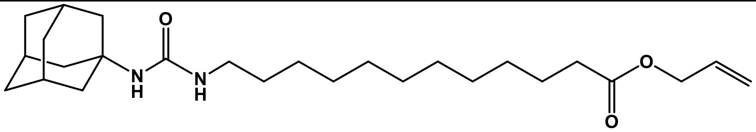	94	42	99	41	90	13
**12**	**G**	**12**	791	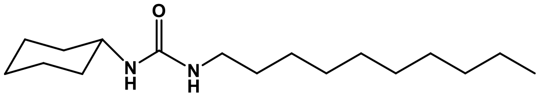	99	64	< 90	90	< 90	48
**12**	**H**	**3**	659	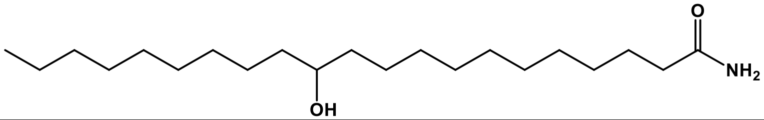	95	83	< 90	36	< 90	16
**12**	**H**	**5**	711	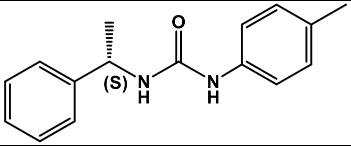	110	91	< 90	54	< 90	13
**12**	**H**	**11**	782	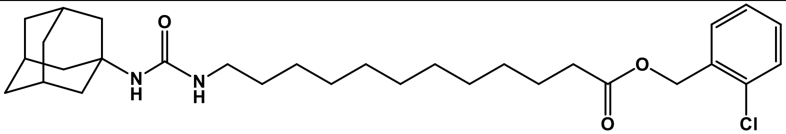	103	20	93	20	90	18
**12**	**H**	**12**	792	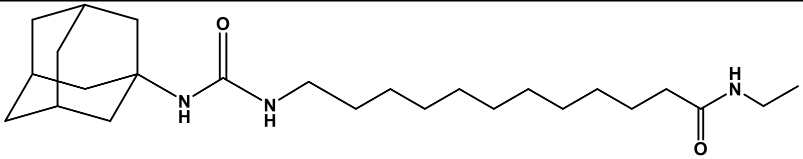	107	85	< 90	98	< 90	79
**13**	**A**	**3**	804	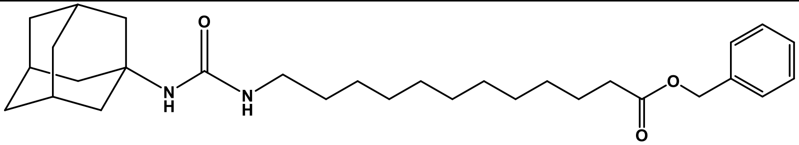	< 90	38	92	54	< 90	31
**13**	**A**	**4**	830	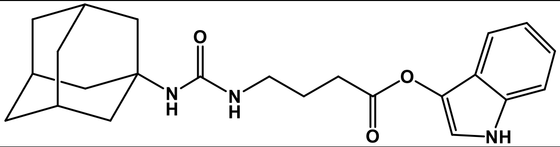	101	86	< 90	96	< 90	68
**13**	**A**	**6**	861	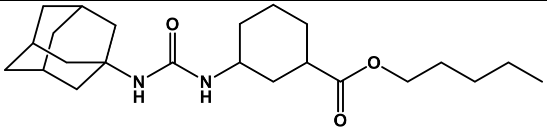	< 90	64	93	84	< 90	55

**13**	**A**	**12**	948	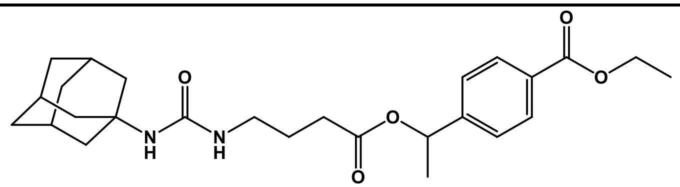	96	69	< 90	59	< 90	75
**13**	**B**	**5**	847	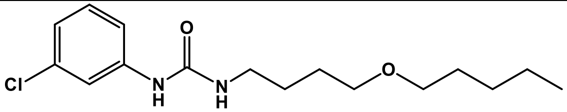	93	82	< 90	84	< 90	44
**13**	**C**	**5**	849	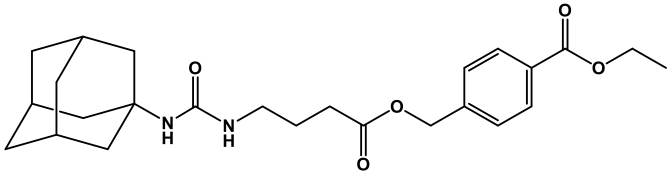	< 90	91	< 90	100	90	95
**13**	**C**	**8**	905	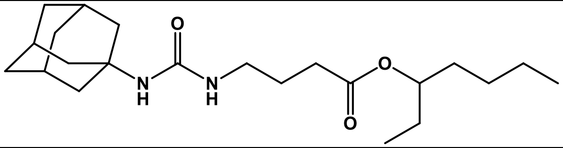	99	83	94	68	< 90	60
**13**	**C**	**11**	941	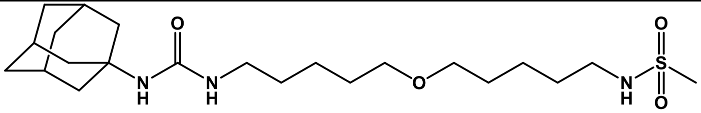	95	84	93	91	< 90	94
**13**	**D**	**6**	866	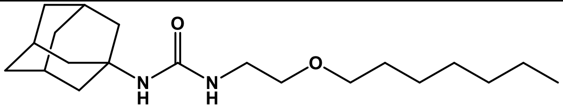	90	80	< 90	89	< 90	57
**13**	**D**	**8**	906	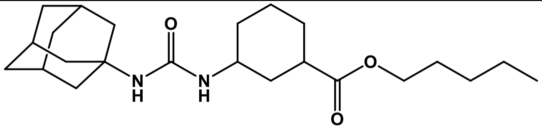	102	84	99	84	90	78
**13**	**E**	**2**	799	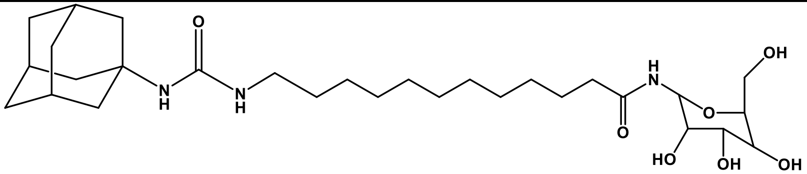	96	85	93	102	90	75
**13**	**E**	**6**	867	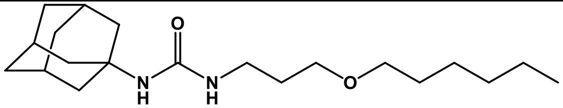	102	86	104	99	90	85

**13**	**E**	**7**	875	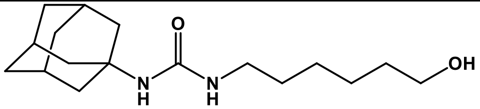	98	73	< 90	58	< 90	37
**13**	**E**	**8**	908	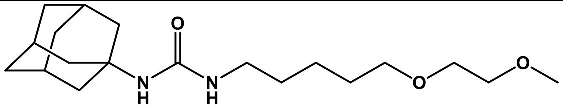	98	90	91	83	< 90	69
**13**	**E**	**11**	943	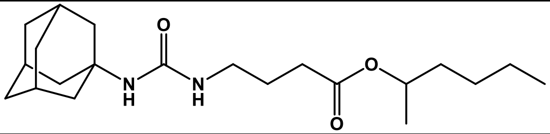	100	89	103	103	< 90	87
**13**	**E**	**12**	956	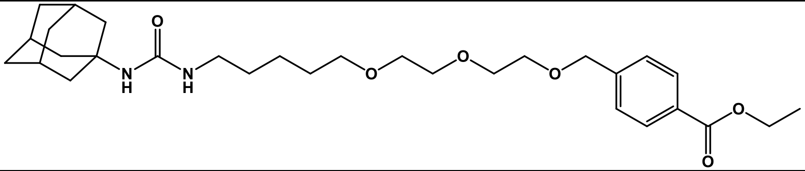	101	90	98	96	< 90	64
**13**	**F**	**6**	868	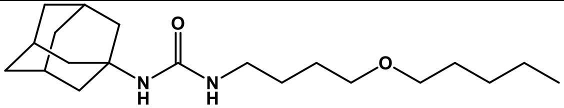	105	90	105	102	90	78
**13**	**F**	**8**	909	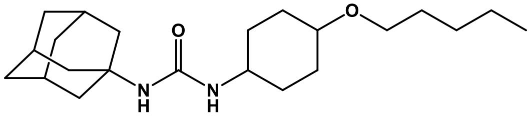	97	80	< 90	94	< 90	88
**13**	**F**	**11**	944	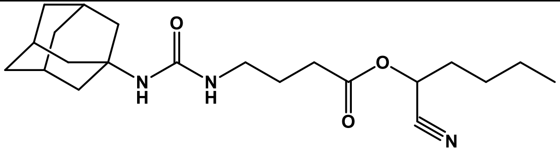	103	89	104	97	90	56
**13**	**F**	**12**	959	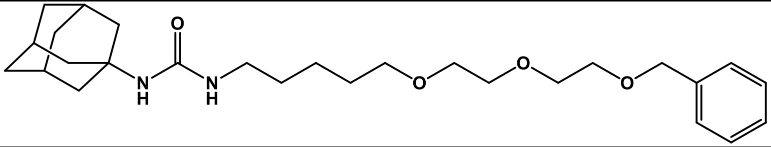	98	76	< 90	81	< 90	44
**13**	**G**	**6**	869	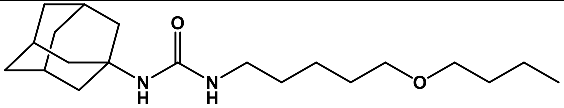	106	90	110	99	< 90	67
**13**	**G**	**7**	882	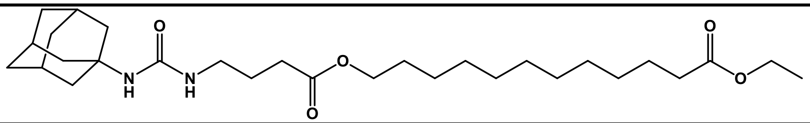	92	30	93	34	90	63

**13**	**G**	**8**	910	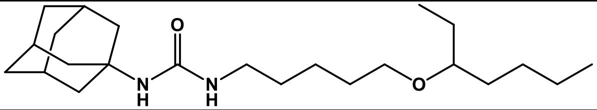	98	74	< 90	78	< 90	54
**13**	**G**	**11**	945	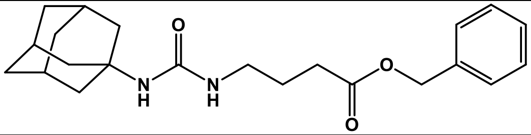	99	89	102	98	< 90	96
**13**	**G**	**12**	960	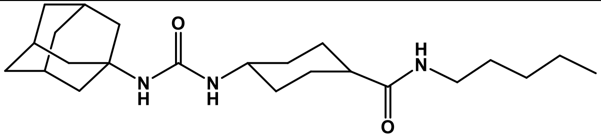	95	89	97	94	< 90	70
**13**	**H**	**5**	860	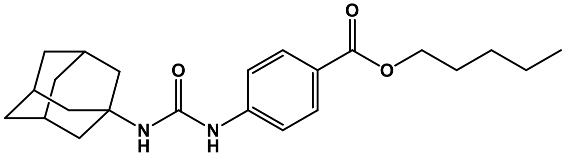	98	75	< 90	58	< 90	63
**13**	**H**	**6**	870	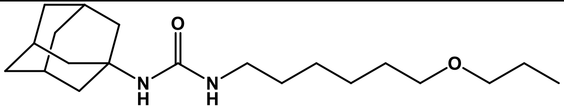	117	88	109	100	< 90	74
**13**	**H**	**11**	947	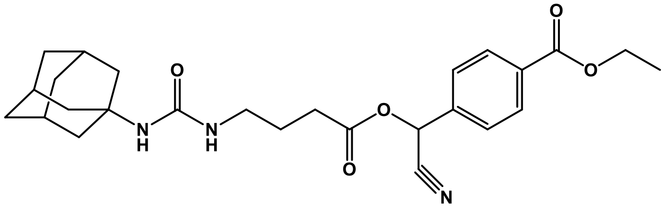	103	77	96	76	< 90	70
**14**	**A**	**4**	982	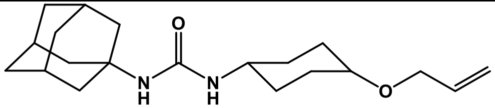	97	89	91	98	90	80
**14**	**A**	**6**	1003	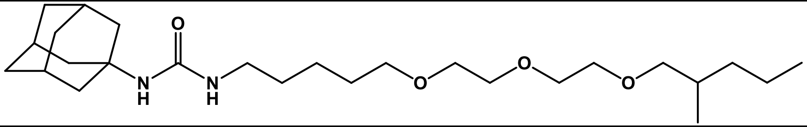	104	86	< 90	80	< 90	60
**14**	**A**	**11**	1744	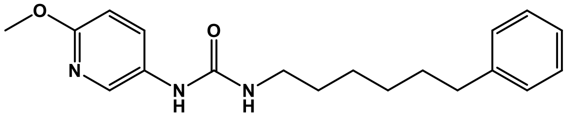	102	79	< 90	45	< 90	37

**14**	**A**	**12**	1752	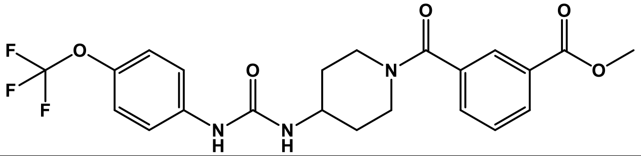	121	92	94	94	< 90	35
**14**	**B**	**3**	973	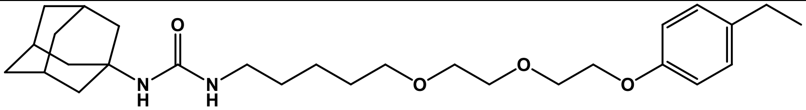	97	89	< 90	75	< 90	41
**14**	**B**	**6**	1004	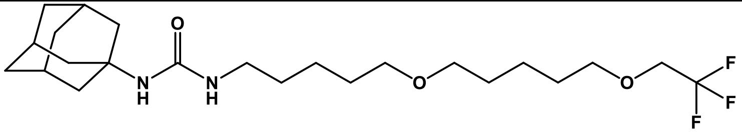	104	88	97	100	90	80
**14**	**B**	**8**	1030	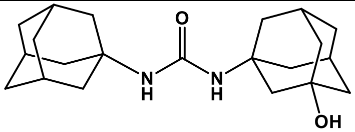	94	74	< 90	60	< 90	32
**14**	**C**	**3**	974	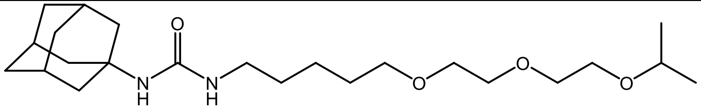	104	87	91	98	< 90	66
**14**	**C**	**4**	986	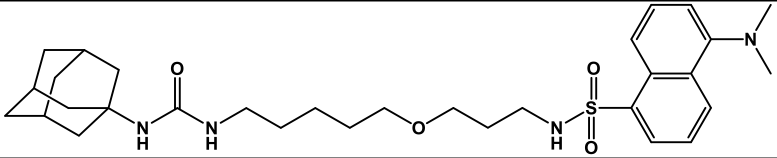	94	64	< 90	44	< 90	35
**14**	**C**	**7**	1021	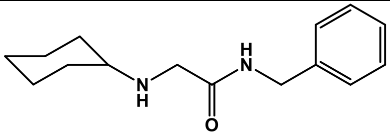	92	88	< 90	47	< 90	32
**14**	**C**	**8**	1031	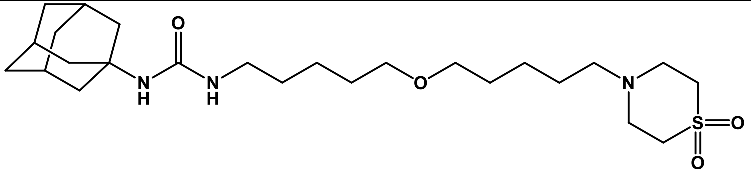	102	65	< 90	23	< 90	27
**14**	**C**	**9**	1039	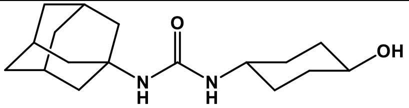	102	88	< 90	68	< 90	29
**14**	**C**	**10**	1049	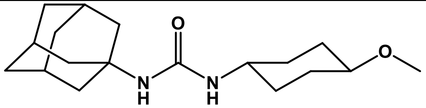	107	90	< 90	85	< 90	56

**14**	**C**	**11**	1746	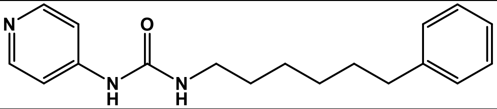	98	47	< 90	28	< 90	24
**14**	**D**	**6**	1009	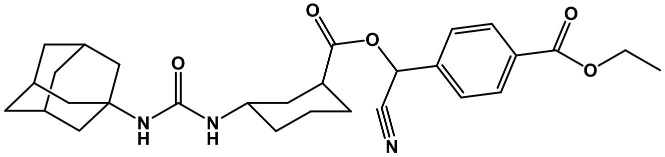 Trans	103	49	< 90	28	< 90	28
**14**	**D**	**7**	1023	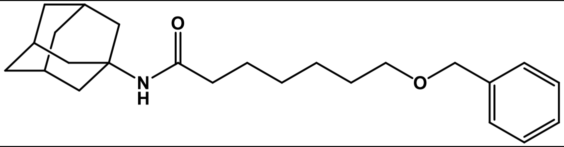	97	78	< 90	58	< 90	19
**14**	**D**	**8**	1032	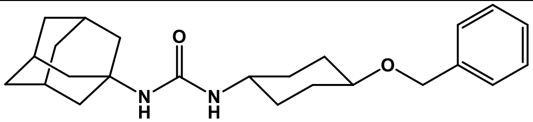	97	78	< 90	70	< 90	46
**14**	**D**	**9**	1040	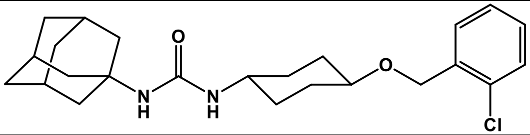	94	73	< 90	49	< 90	71
**14**	**D**	**11**	1747	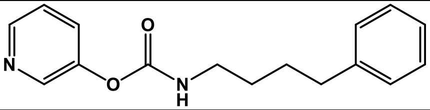	111	90	< 90	75	< 90	40
**14**	**E**	**2**	967	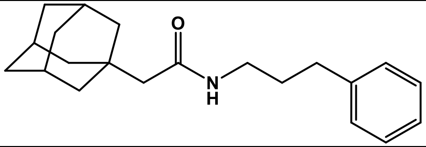	99	91	< 90	63	< 90	69
**14**	**E**	**7**	1024	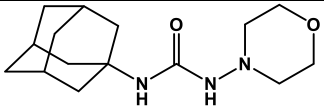	98	42	< 90	22	< 90	65
**14**	**E**	**9**	1041	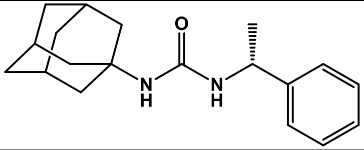	93	32	< 90	12	< 90	-16

**14**	**E**	**11**	1748	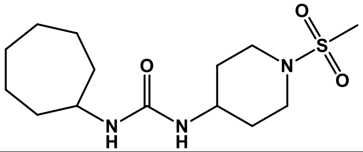	124	85	< 90	35	< 90	34
**14**	**E**	**12**	1756	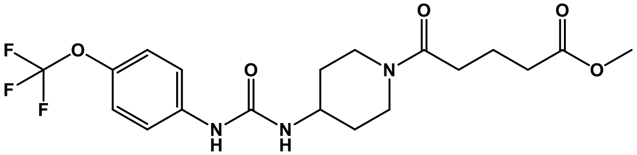	122	86	< 90	43	< 90	-1
**14**	**F**	**7**	1025	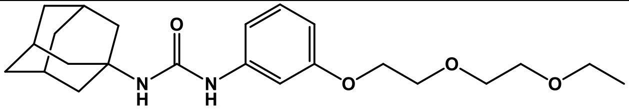	110	92	101	99	90	87
**14**	**F**	**9**	1042	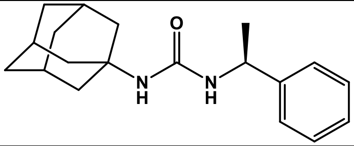	110	91	< 90	83	< 90	64
**14**	**F**	**10**	1741	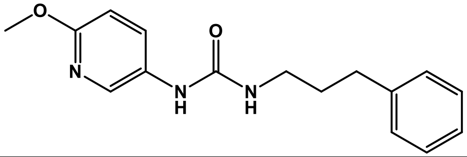	92	86	< 90	38	< 90	8
**14**	**F**	**11**	1749	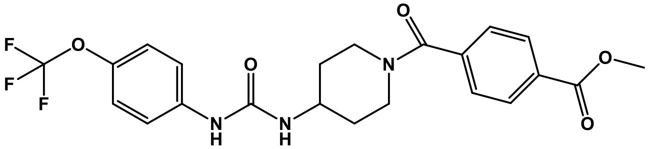	115	91	100	94	< 90	49
**14**	**F**	**12**	1757	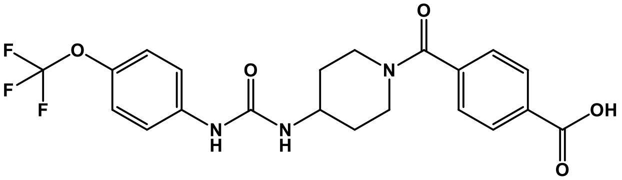	104	92	< 90	87	< 90	58

**14**	**G**	**5**	1001	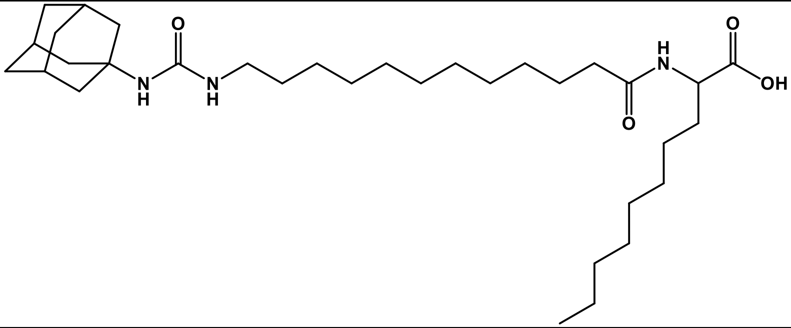	92	93	100	95	90	77
**14**	**G**	**7**	1026	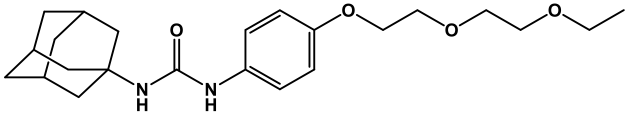	101	87	98	94	90	57
**14**	**H**	**4**	991	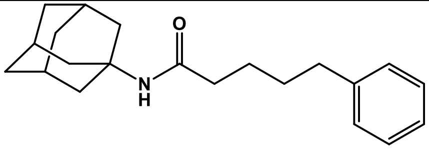	97	72	< 90	35	< 90	29
**14**	**H**	**5**	1002	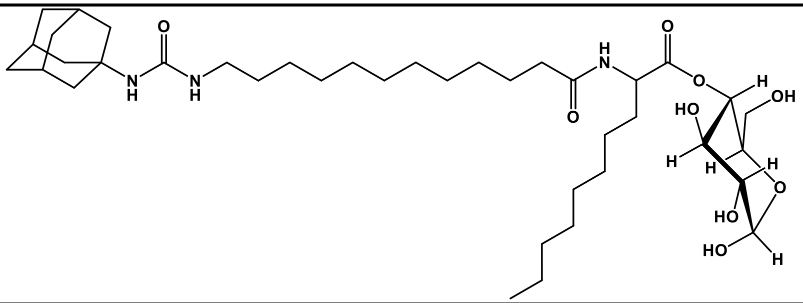	96	41	< 90	36	< 90	18
**14**	**H**	**6**	1017	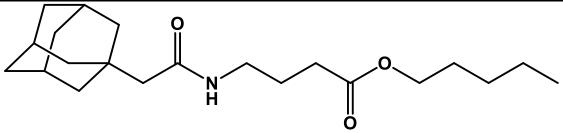	116	92	< 90	78	< 90	43
**14**	**H**	**8**	1036	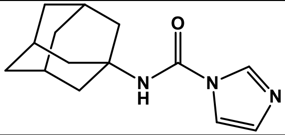	92	66	< 90	35	< 90	41
**14**	**H**	**9**	1045	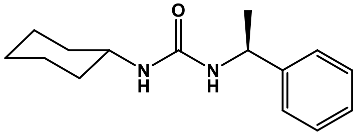	118	93	< 90	77	< 90	68

**14**	**H**	**10**	1743	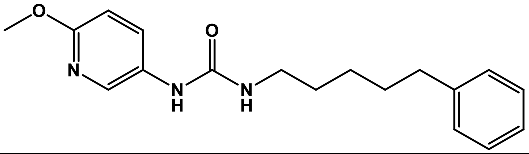	113	74	< 90	60	< 90	32
**14**	**H**	**11**	1751	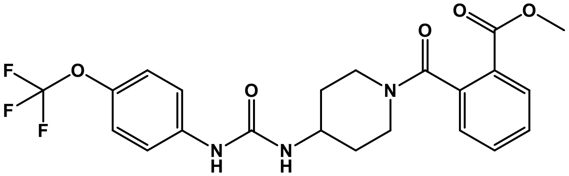	123	92	< 90	90	< 90	14
**14**	**H**	**12**	1759	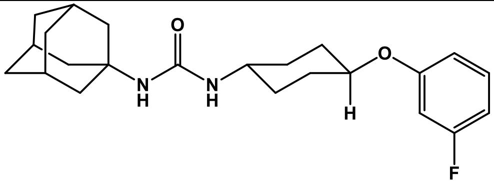	118	89	99	66	90	85
**15**	**A**	**9**	1816	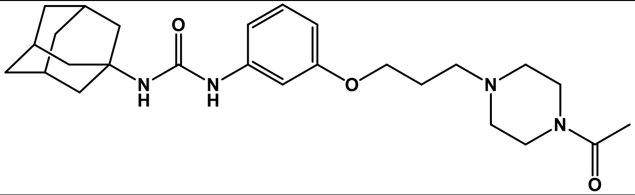	97	87	103	89	91	80
**15**	**B**	**2**	1761	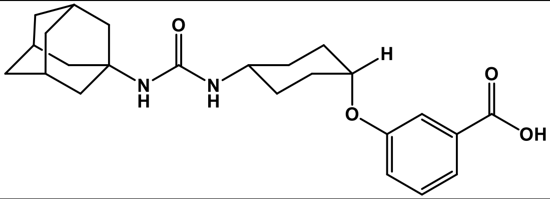	99	91	95	99	90	91
**15**	**B**	**6**	1793	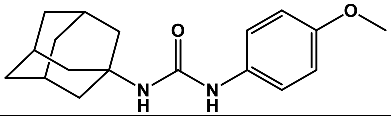	93	91	< 90	87	< 90	54
**15**	**C**	**2**	1762	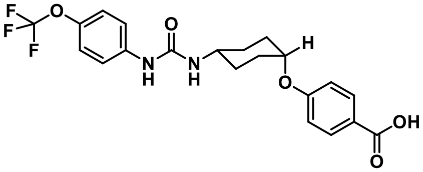	101	94	93	99	< 90	89
**15**	**C**	**4**	1778	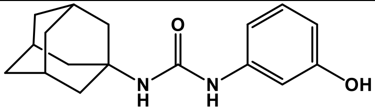	90	79	< 90	61	< 90	31

**15**	**C**	**5**	1786	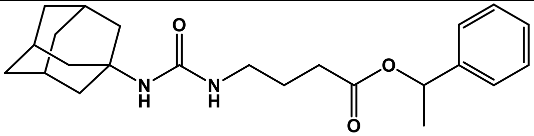	100	95	< 90	99	92	80
**15**	**D**	**3**	1771	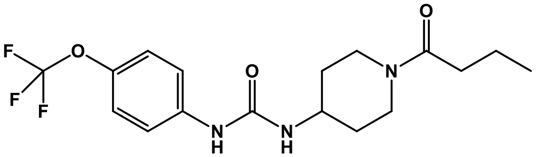	100	91	< 90	67	< 90	-7
**15**	**D**	**4**	1779	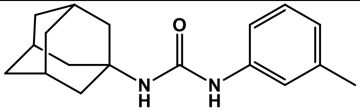	97	85	< 90	71	< 90	73
**15**	**D**	**5**	1787	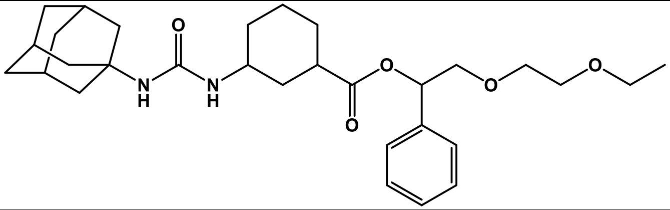	95	81	< 90	49	< 90	64
**15**	**D**	**11**	1835	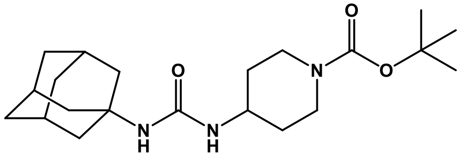	103	89	93	99	93	86
**15**	**D**	**12**	1843	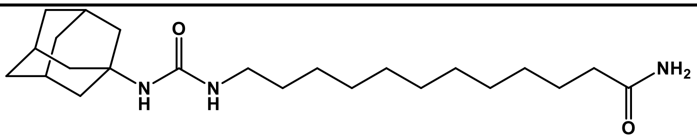	105	91	12	98	99	85
**15**	**E**	**9**	1820	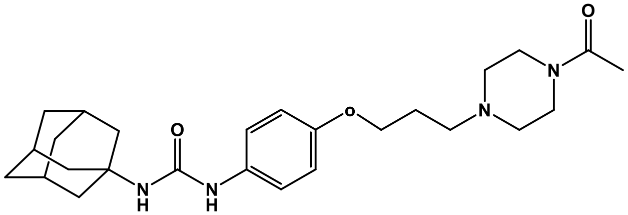	94	92	< 90	77	< 90	54
**15**	**E**	**10**	1828	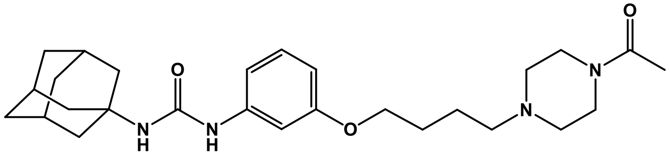	100	95	93	98	91	81

**15**	**E**	**12**	1844	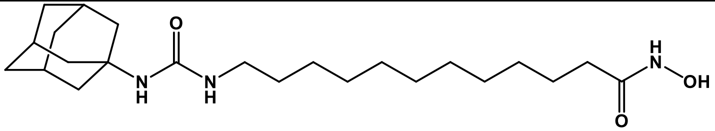	< 90	90	102	99	91	80
**15**	**F**	**7**	1805	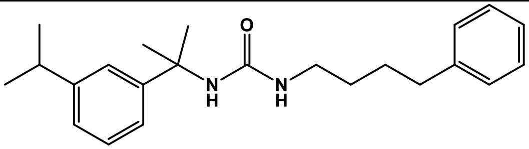	98	103	< 90	95	< 90	72
**15**	**F**	**12**	1845	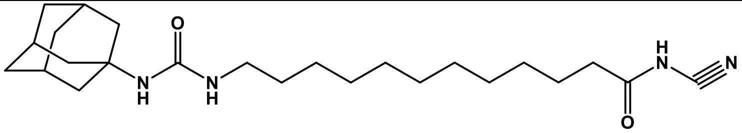	100	88	101	104	95	83
**15**	**G**	**2**	1766	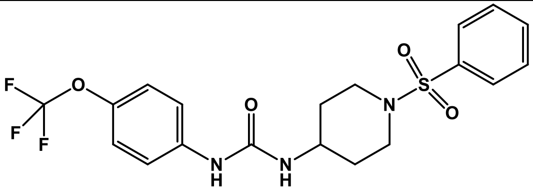	103	85	< 90	78	< 90	35
**15**	**G**	**4**	1782	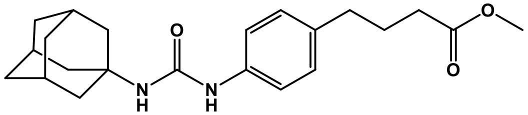	93	85	< 90	92	< 90	76
**15**	**G**	**7**	1806	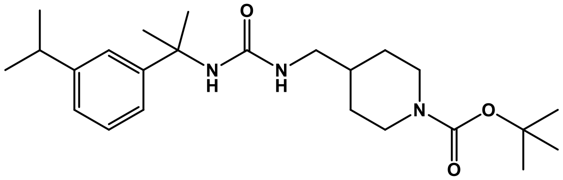	103	96	102	90	< 90	81
**15**	**H**	**5**	1791	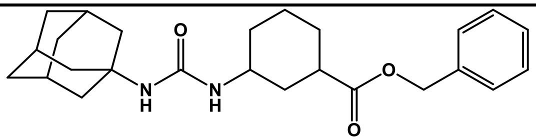	102	84	91	75	92	74
**17**	**H**	**9**	Y4-08	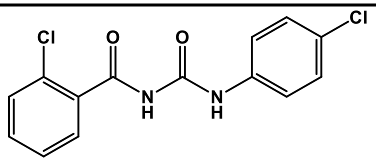	94	19	< 90	8	< 90	3
**17**	**H**	**12**	Y4-33	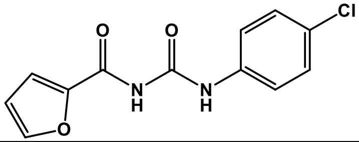	94	12	< 90	12	< 90	1

**18**	**B**	**11**	1734	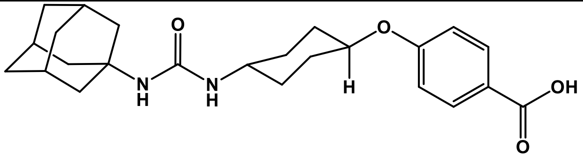	99	90	96	97	90	89
**18**	**D**	**11**	1736	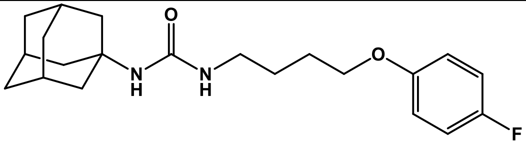	97	90	96	98	< 90	67
**18**	**E**	**11**	1737	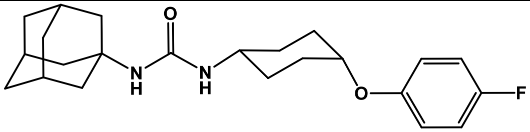	97	86	96	80	92	80
**19**	**A**	**4**	1875	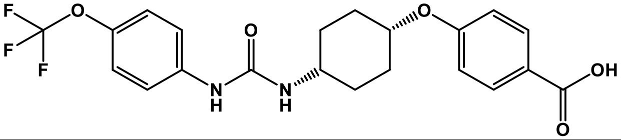	105	91	99	100	91	76
**19**	**A**	**6**	1891	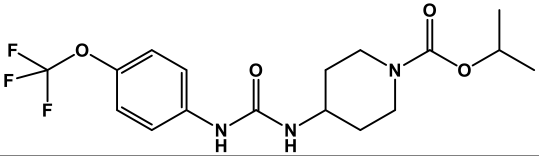	96	94	< 90	73	< 90	37
**19**	**B**	**3**	1868	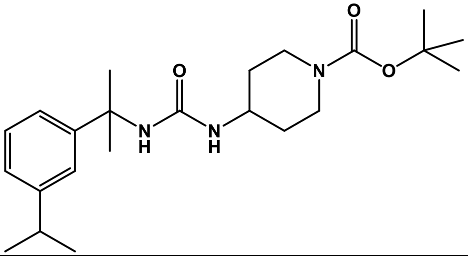	94	92	< 90	79	< 90	51
**19**	**B**	**9**	1956	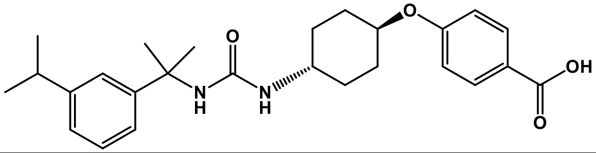	< 90	73	91	100	91	87
**19**	**C**	**4**	1877	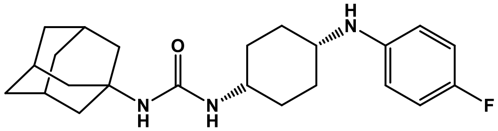	93	88	92	92	90	80

**19**	**D**	**2**	1862	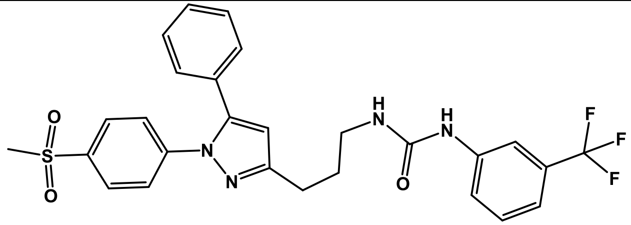	97	90	< 90	64	< 90	29
**19**	**D**	**3**	1870	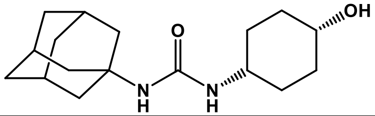	91	61	< 90	33	90	-15
**19**	**F**	**8**	1912	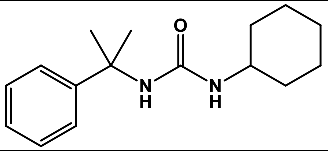	97	82	< 90	27	< 90	9
**19**	**H**	**3**	1874	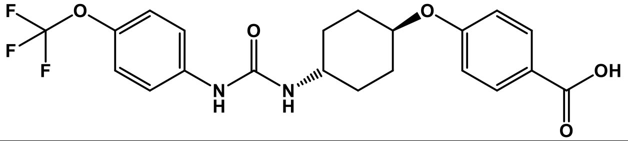	94	89	104	99	90	64
**19**	**H**	**11**	2013	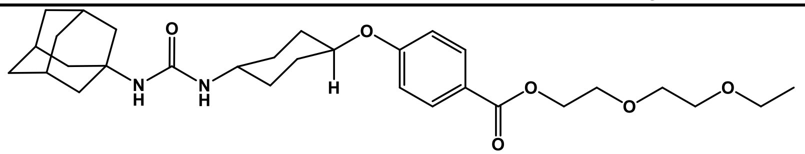	101	74	102	53	90	68
**21**	**H**	**9**	Siduron	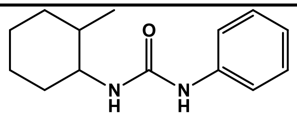	92	81	93	77	< 90	43
**22**	**A**	**6**	2143	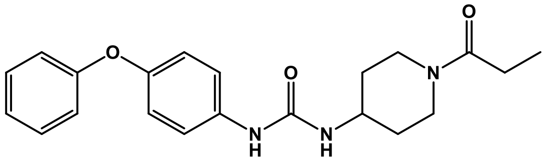	< 90	87	91	83	< 90	35
**22**	**B**	**7**	2186	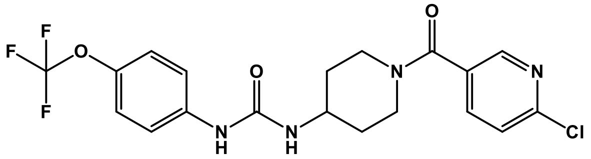	< 90	89		84	92	11

**22**	**C**	**6**	2145	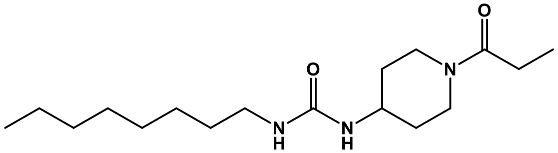	< 90	90	< 90	90	94	82
**22**	**C**	**9**	2225	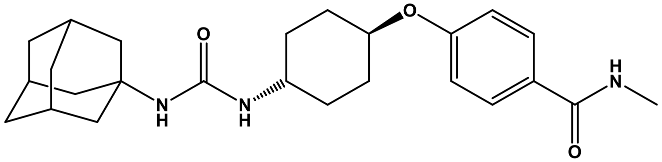	90	88	< 90	102	92	83
**22**	**C**	**10**	2245	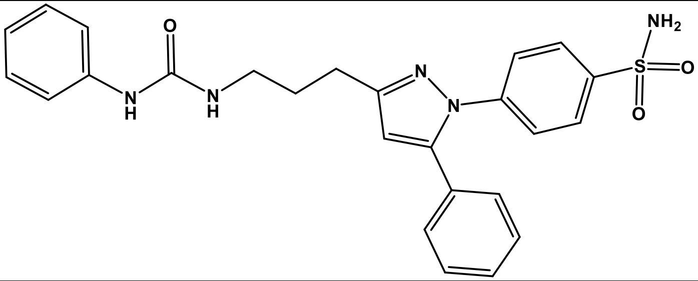	97	85	< 90	83	< 90	38
**22**	**D**	**7**	2190	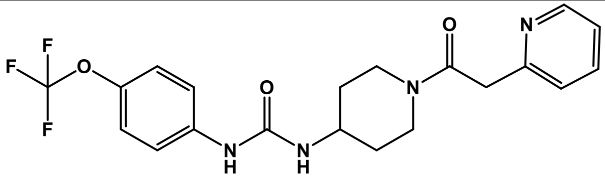	< 90	81	94	34	< 90	9
**22**	**D**	**9**	2227	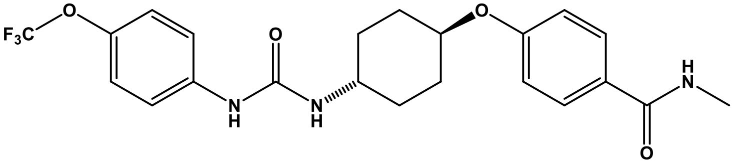	96	91	97	89	< 90	18
**22**	**D**	**10**	2246	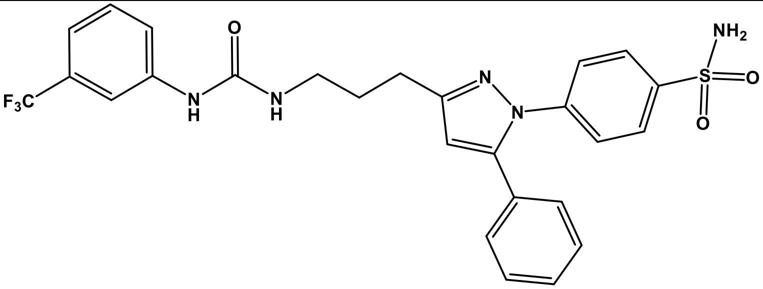	97	90	< 90	76	< 90	55

**22**	**E**	**7**	2191	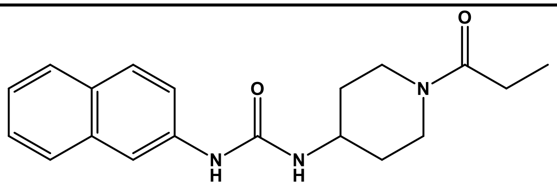	93	92	94	73	< 90	11
**22**	**E**	**9**	2228	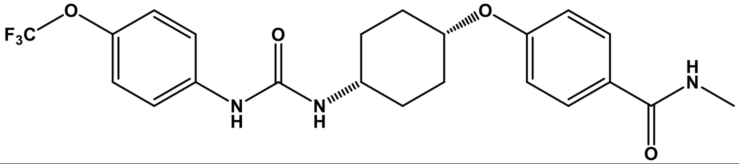	97	94	91	103	94	85
**22**	**E**	**10**	2247	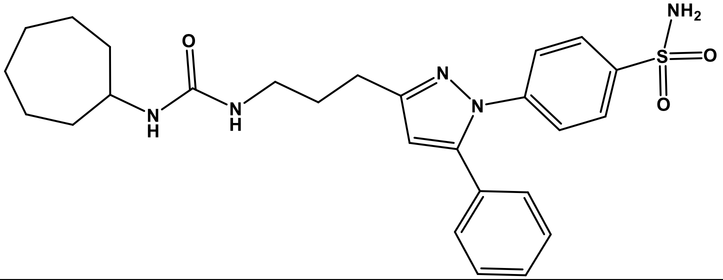	98	95	109	103	93	89
**22**	**E**	**11**	2574	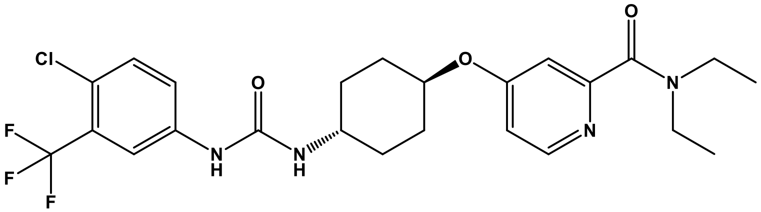	94	90	93	88	90	62
**22**	**F**	**5**	2139	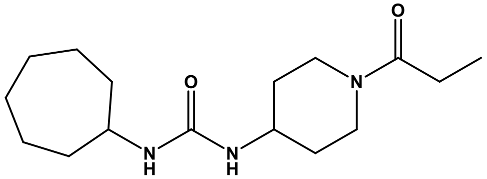	97	83	< 90	51	< 90	50
**22**	**F**	**6**	2156	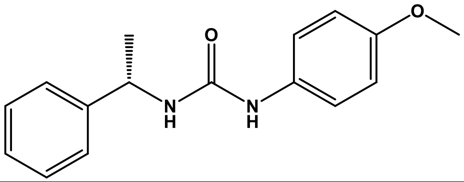	99	90	< 90	43	< 90	10

**22**	**F**	**11**	2575	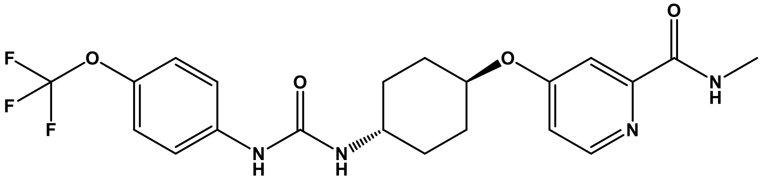	97	93	95	98	< 90	36
**22**	**G**	**6**	2157	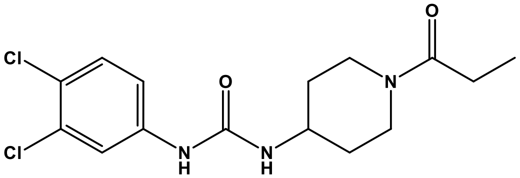	98	93	< 90	55	< 90	38
**22**	**G**	**8**	2208	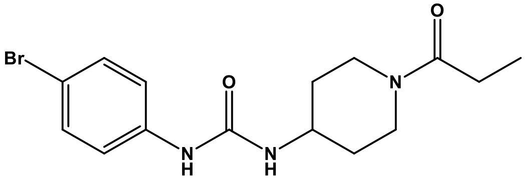	92	91	< 90	35	< 90	7
**22**	**G**	**9**	2278	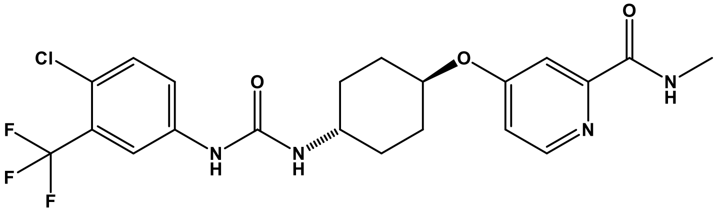	98	96	100	94	92	41
**22**	**H**	**5**	2142	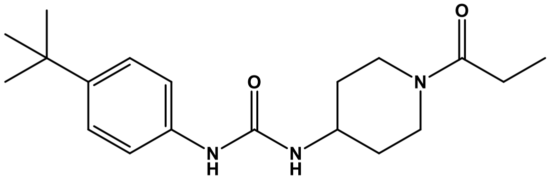	102	92	< 90	30	< 90	-14
**22**	**H**	**9**	2287	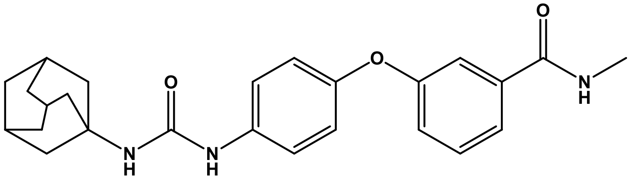	98	84	100	99	90	84

**22**	**H**	**10**	2327	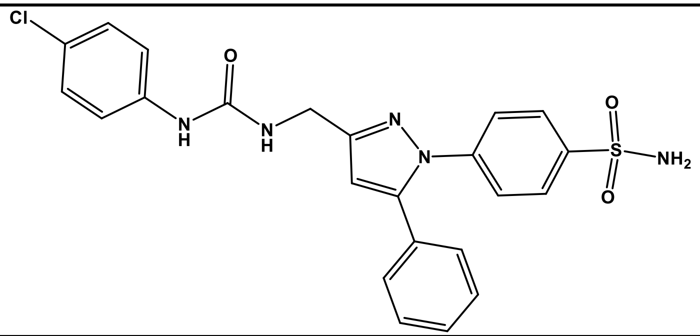	99	83	< 90	83	< 90	32
**22**	**H**	**11**	2578	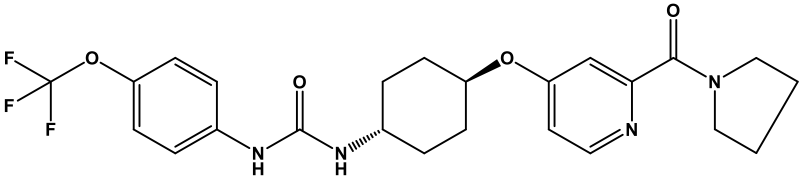	92	92	94	89	< 90	29
**23**	**A**	**2**	2119	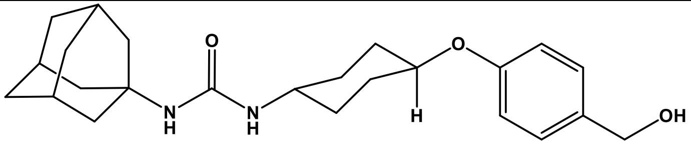	91	90	96	104	< 90	80
**23**	**A**	**5**	2549	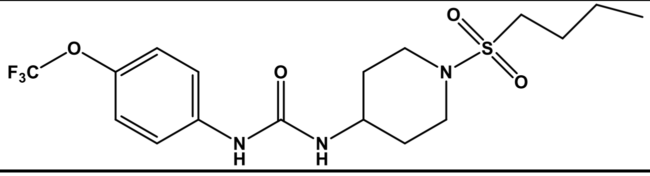	95	89	< 90	63	< 90	27
**23**	**A**	**6**	2728	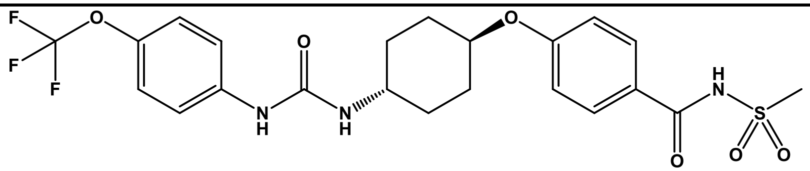	101	92	92	99	90	71
**23**	**A**	**9**	2646	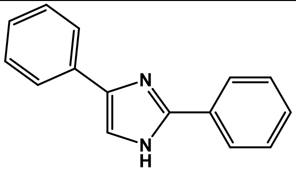	< 90	23	109	12	< 90	-6
**23**	**A**	**12**	2805	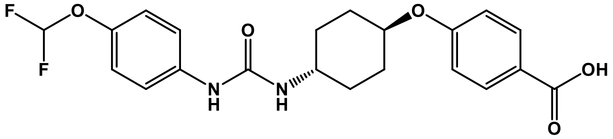	< 90	86	96	99	< 90	73

**23**	**B**	**5**	2214	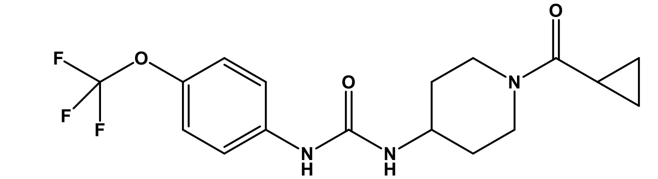	96	89	< 90	56	< 90	16
**23**	**B**	**6**	1686	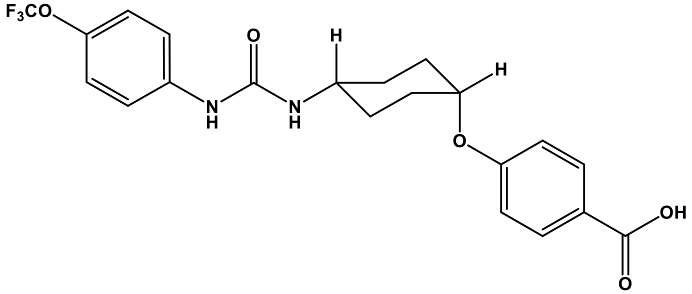	103	91	99	110	90	86
**23**	**C**	**5**	2414	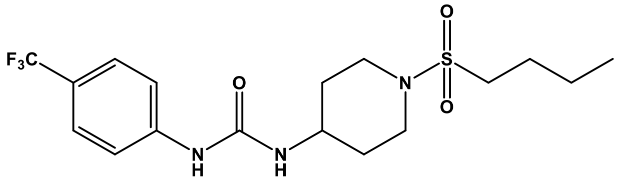	98	90	< 90	53	< 90	24
**23**	**C**	**7**	2736	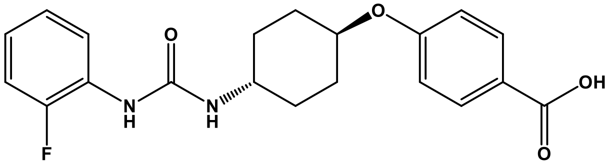	97	66	< 90	58	< 90	43
**23**	**C**	**11**	2827	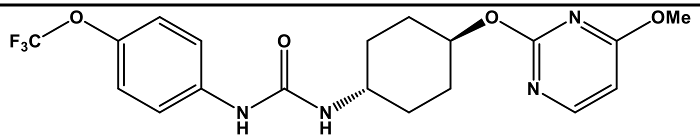	97	93	< 90	99	< 90	40
**23**	**D**	**5**	2634	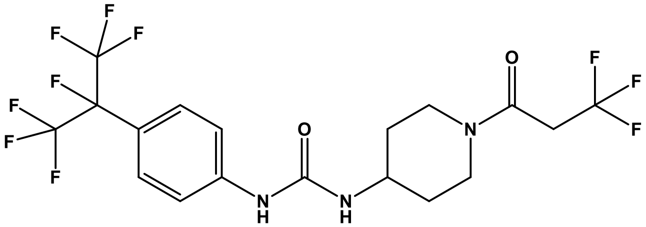	95	85	< 90	86	< 90	9

**23**	**D**	**6**	2472	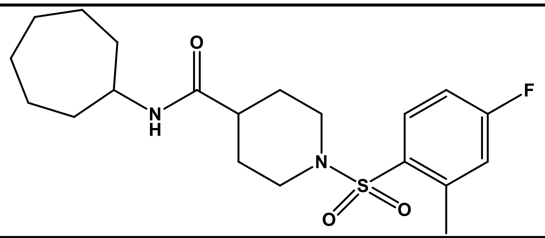	94	95	< 90	44	< 90	-1
**23**	**D**	**7**	2797	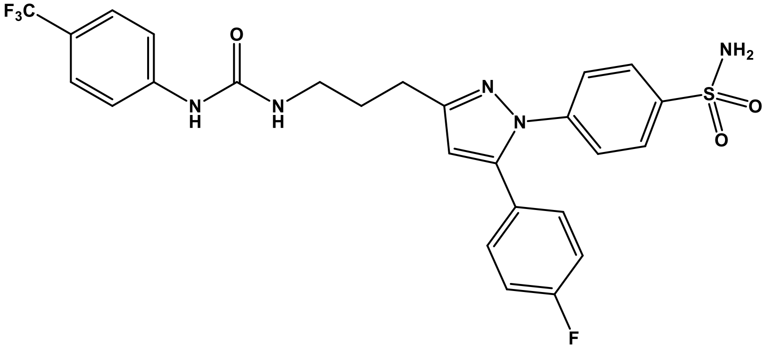	91	88	< 90	93	< 90	18
**23**	**D**	**12**	2810	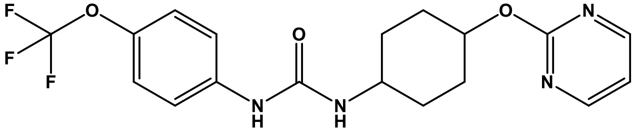	107	87	< 90	84	< 90	13
**23**	**E**	**2**	2804	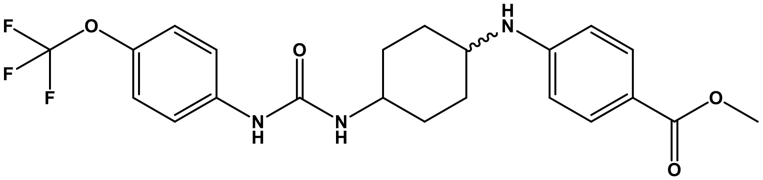	90	66	< 90	33	< 90	7
**23**	**E**	**5**	2415	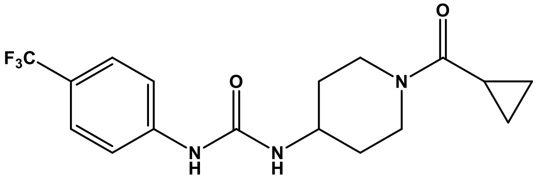	96	93	< 90	64	< 90	15
**23**	**E**	**7**	2901	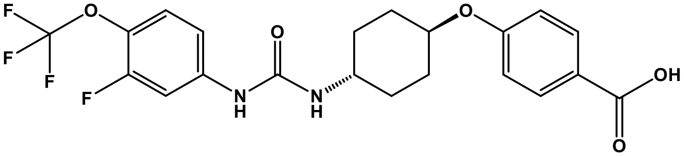	102	96	106	101	91	82

**23**	**F**	**5**	2633	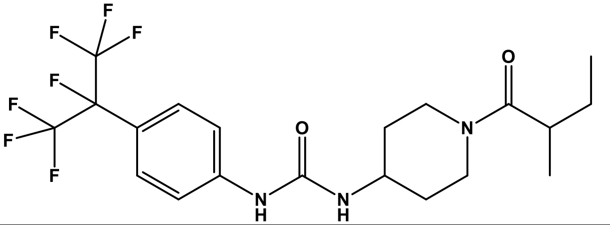	105	95	97	95	< 90	57
**23**	**F**	**7**	2803	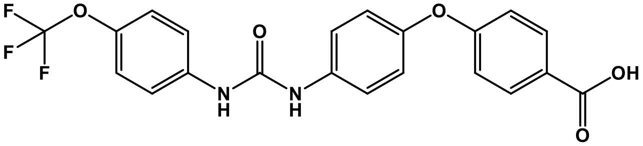	< 90	88	109	98	< 90	56
**23**	**F**	**11**	2709	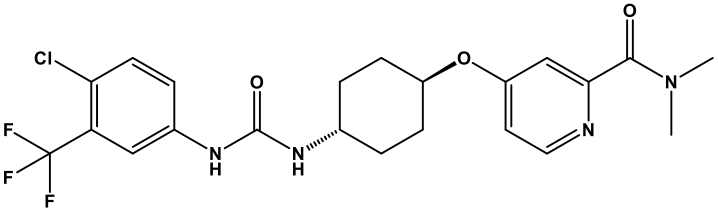	107	94	99	90	< 90	58
**23**	**F**	**12**	2221	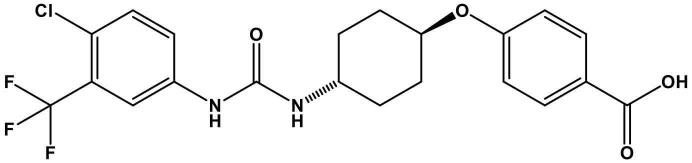	106	93	100	104	97	84
**23**	**G**	**6**	2904	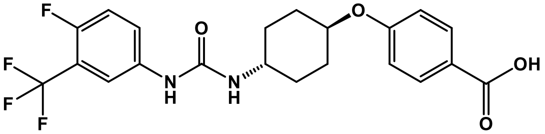	99	88	99	103	91	83
**23**	**G**	**7**	2902	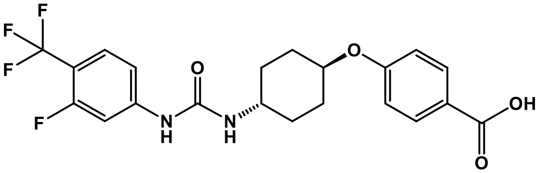	102	84	100	102	93	72
**23**	**G**	**12**	2825	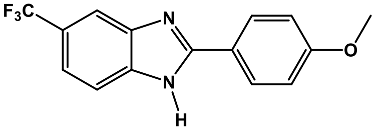	97	86	< 90	29	< 90	24
**23**	**H**	**11**	2806	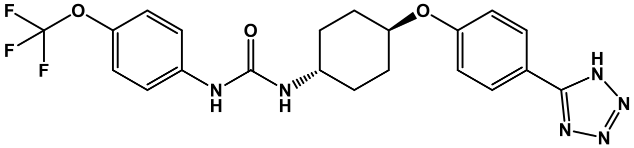	105	92	100	100	92	84

**24**	**A**	**2**	2487	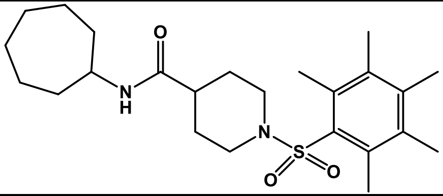	90	92	< 90	97	< 90	76
**24**	**B**	**6**	2535	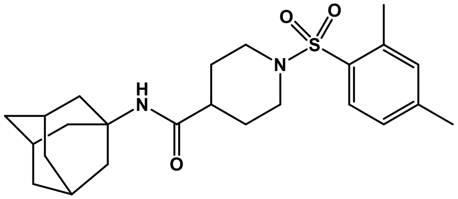	90	88	< 90	72	< 90	78
**24**	**C**	**5**	2451	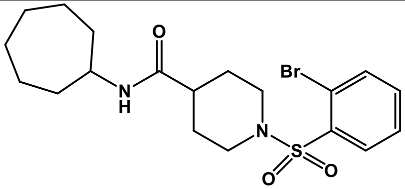	97	92	98	95	< 90	70
**24**	**C**	**9**	SP-I-191C	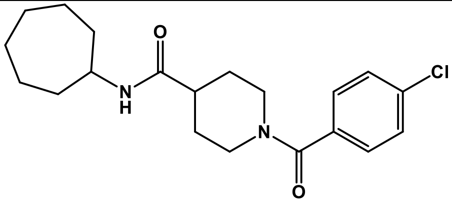	95	67	< 90	37	< 90	49
**24**	**C**	**12**	2567	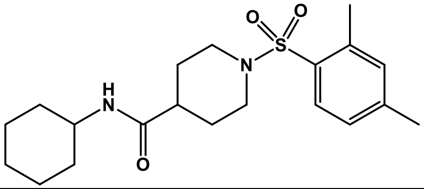	94	87	< 90	96	< 90	63
**24**	**D**	**2**	2482	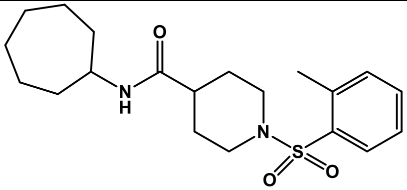	92	85	92	105	< 90	65

**24**	**D**	**6**	2447	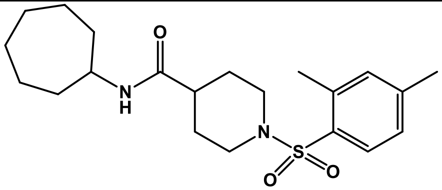	96	95	< 90	98	< 90	82
**24**	**E**	**7**	2536	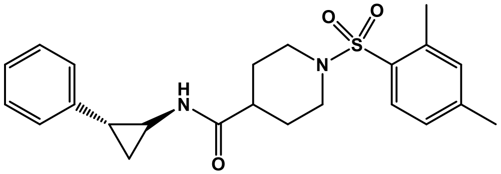	97	92	< 90	92	< 90	6
**24**	**E**	**8**	2490	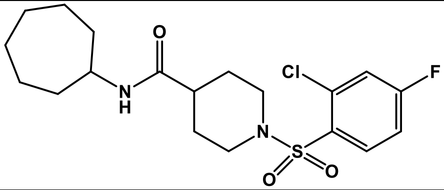	95	90	< 90	82	< 90	51
**24**	**E**	**10**	2343	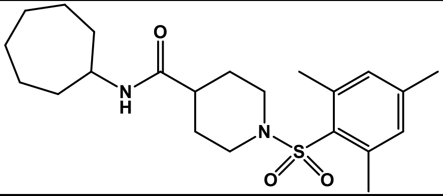	102	92	97	96	< 90	78
**24**	**F**	**6**	2492	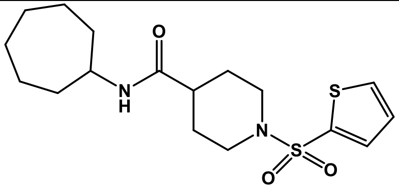	90	72	< 90	51	< 90	11
**24**	**G**	**3**	2472	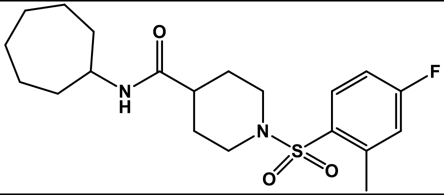	95	91	< 90	80	< 90	37
**25**	**C**	**11**	MDC-I-157	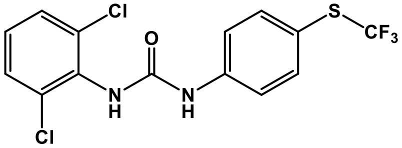	< 90	59	92	34	< 90	5

**25**	**D**	**5**	MDC-I-169	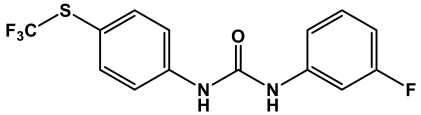	90	66	< 90	69	< 90	6
**25**	**D**	**7**	MDC-I-177	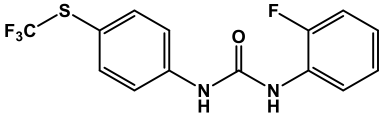	96	84	< 90	78	< 90	11
**25**	**E**	**11**	MDC-I-219	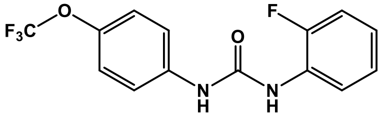	92	90	< 90	93	< 90	18
**25**	**F**	**9**	MDC-I-239	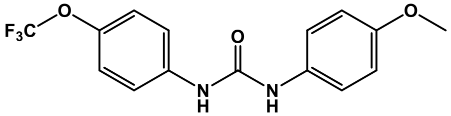	94	75	< 90	23	< 90	7
**25**	**G**	**3**	MDC-I-249	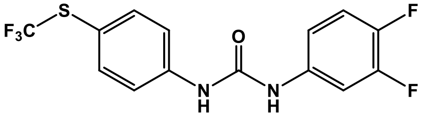	102	93	< 90	93	< 90	34
**25**	**G**	**4**	MDC-I-251	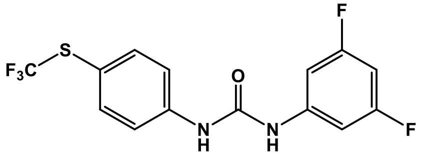	103	92	96	86	93	38
**25**	**G**	**6**	MDC-I-255	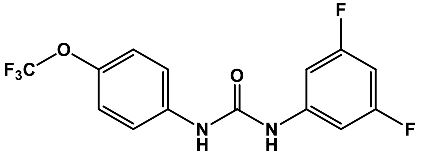	94	76	< 90	59	< 90	0
**25**	**G**	**7**	MDC-I-257	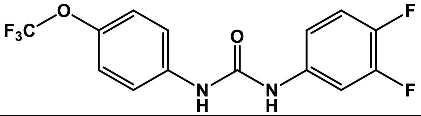	94	62	< 90	47	< 90	6
**25**	**G**	**8**	MDC-I-259	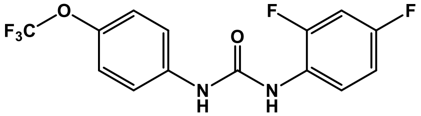	92	62	< 90	83	< 90	4
**25**	**G**	**10**	MDC-I-263	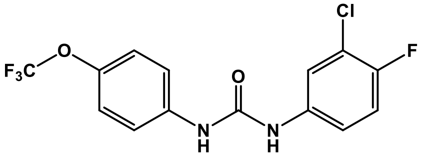	97	69	< 90	14	< 90	3

**25**	**G**	**12**	MDC-I-267	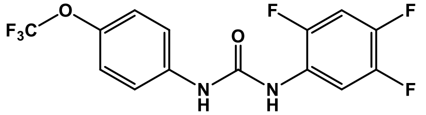	98	88	< 90	79	< 90	16
**25**	**H**	**10**	MDC-2-3	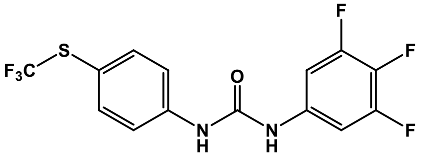	105	93	< 90	87	< 90	24
**26**	**B**	**7**	MDC-2-49	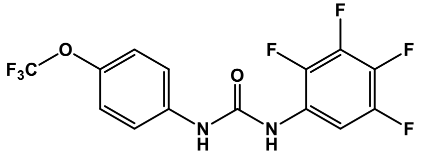	92	92	< 90	93	< 90	24
**26**	**B**	**8**	MDC-2-55	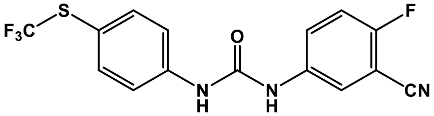	93	92	< 90	64	< 90	33
**26**	**C**	**6**	VMS-3-223	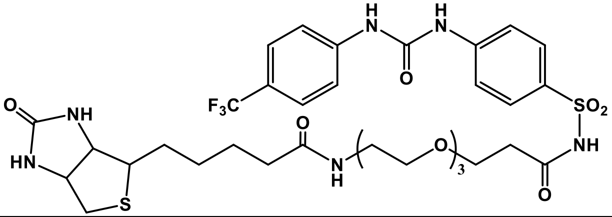	98	94	93	92	< 90	-10
**26**	**C**	**9**	VSM-4-97	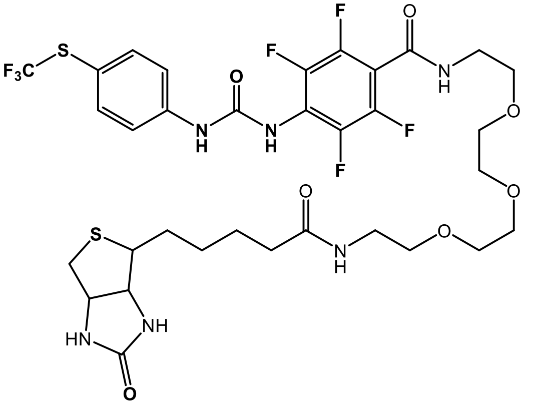	92	85	< 90	39	< 90	6

**26**	**D**	**10**	2571	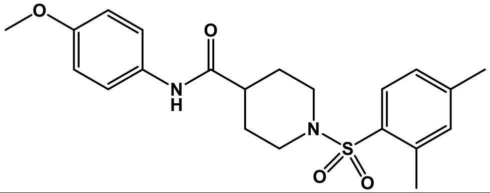	92	72	< 90	54	< 90	27
**26**	**E**	**8**	2595	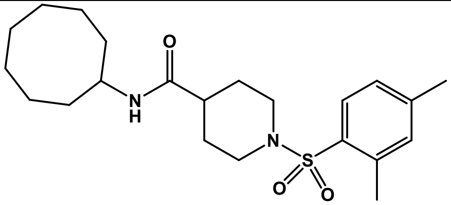	100	89	96	99	98	95
**26**	**E**	**9**	2447	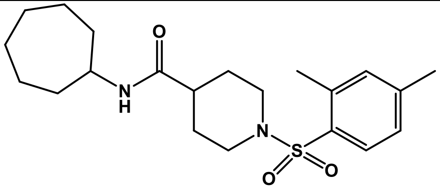	92	92	91	97	102	88
**26**	**E**	**12**	2593	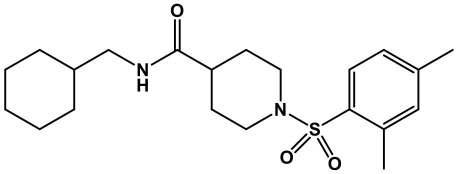	103	97	98	101	109	88
**26**	**F**	**5**	2854	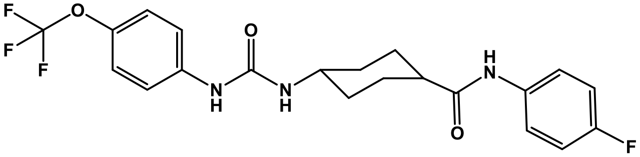	95	95	< 90	69	< 90	12

Table S2: IC_{50}s for the positive hits after the secondary screening.

EHI #	maker #	Structure	Horse sEH	Cat sEH	Dog sEH
			IC_{50} (nM)		
**192**	BH-147-10-01	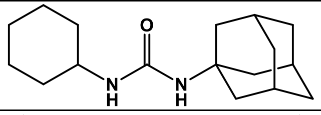	0.4	2.0	3.4
**360**	MG-XXXII-69B	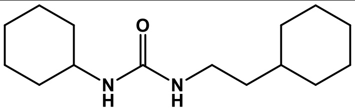	0.4	3.8	9.6
**438**	MG-XXXIII-37A	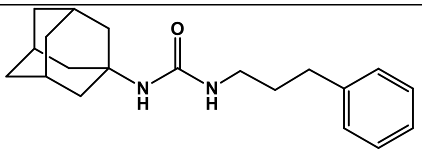	0.9	2.7	2.5
**700**	Cri-76-7	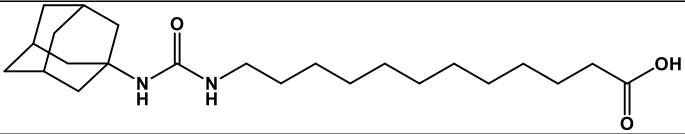	0.4	0.4	0.4
**789**	KIH-237-71	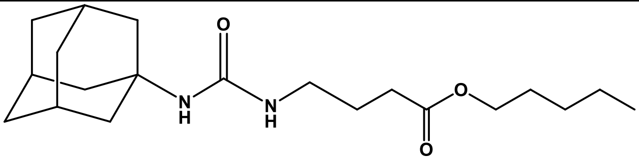	11.1	25.6	51.7
**849**	KIH-258-55	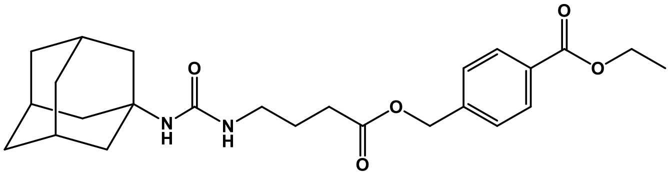	8.9	16.9	34.9
**867**	KIH-258-96	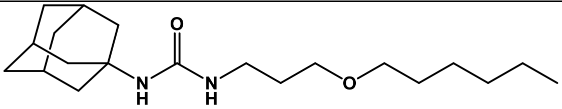	6.5	13.5	31.
**909**	KIH-271-55 B	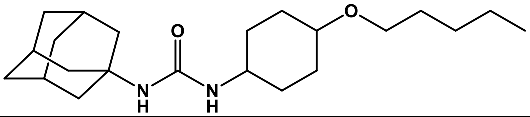	17.1	29.7	23.5
**941**	KIH-271-70	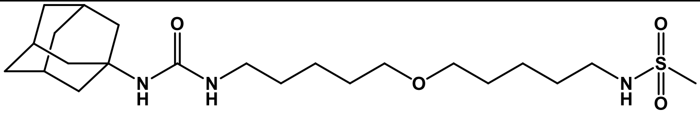	8.4	18.0	26.0
**943**	KIH-271-74	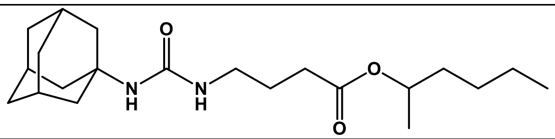	6.5	15.5	16.2
**945**	KIH-271-81	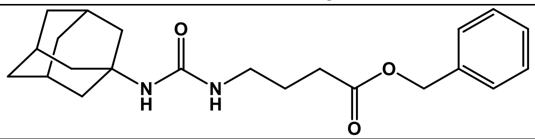	4.5	19.6	34.1
**982**	KIH-284-32	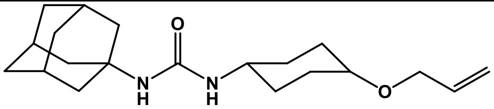	5.5	12.8	5.9

**1004**	KIH-284-51	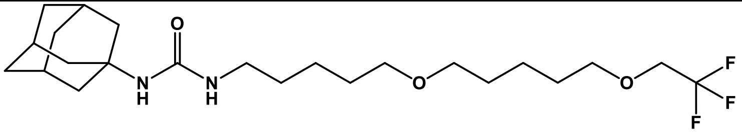	7.2	22.4	24.5
**1025**	KIH-284-79	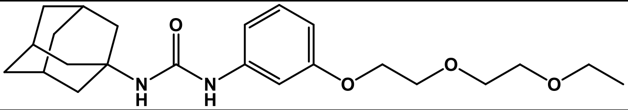	4.2	12.1	10.7
**1078**	SHH-01-062	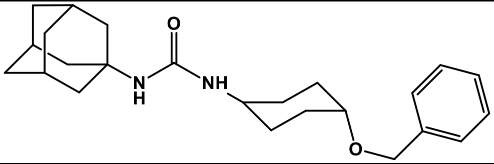	0.4	2.3	0.8
**1141**	SHH02-044	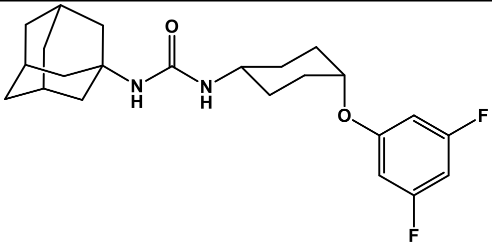	0.4	0.4	1.5
**1203**	pdj-v-82b	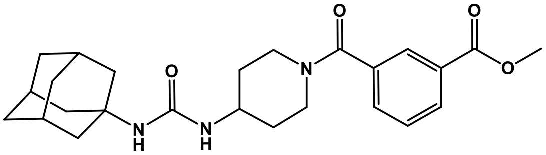	10.5	13.6	2.0
**1242**	SHH-CC-1-10	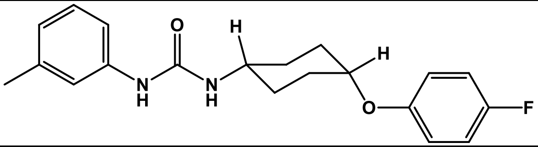	3.8	2.5	12.7
**1254**	SHH-CC-1-22	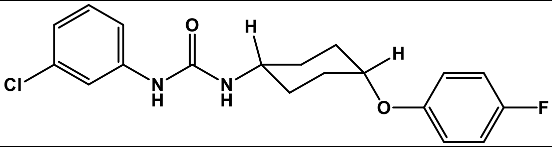	2.8	4.9	14.2
**1270**	SHH-CC-1-38	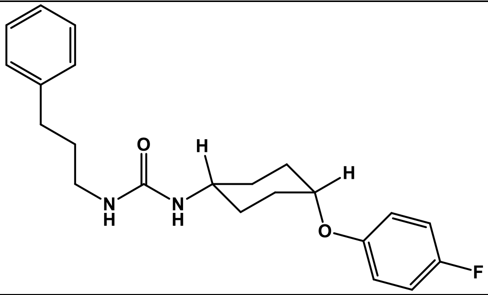	1.9	2.6	8.8
**1271**	SHH-CC-1-39	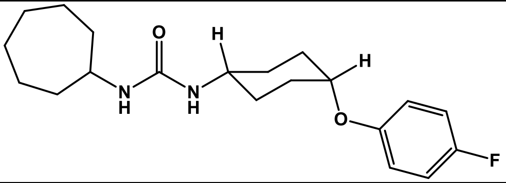	23	2.2	7.3
**1318**	SHH-CC-2-38	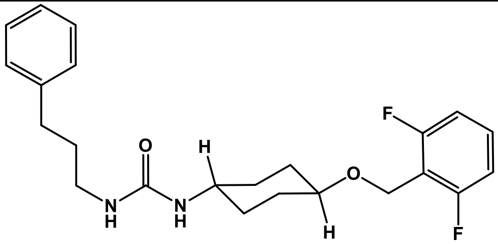	2.4	2.5	13.4

**1471**	SHH02-090	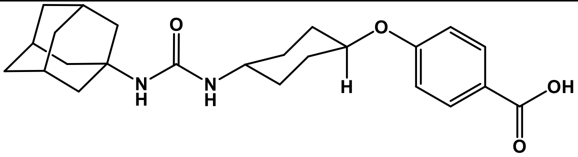	0.4	1.0	0.4
**1565**	SHH-03-001	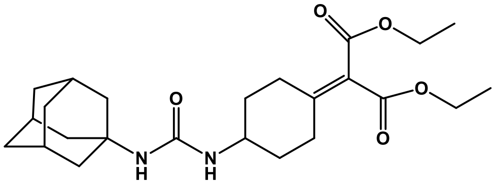	1.2	0.7	1.0
**1620**	KIH-320-99	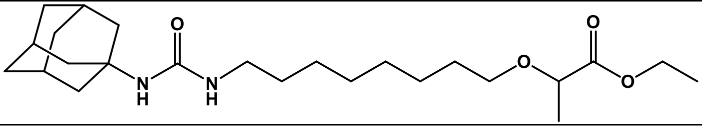	3.5	19.1	18.3
**1621**	KIH-320-96	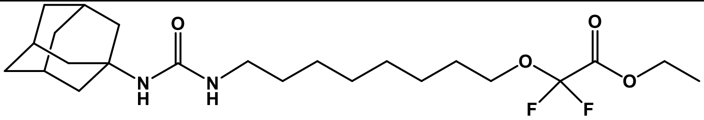	4.8	21.6	23.3
**1663**	TK-32	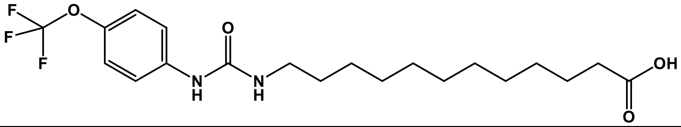	11.7	9.6	1.7
**1675**	SHH-03-042	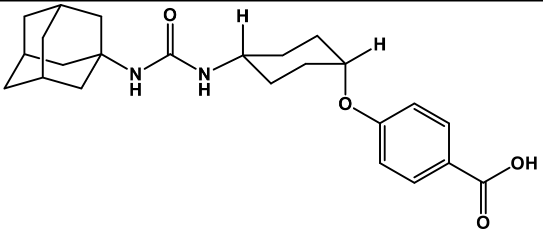	0.4	0.4	0.5
**1686**	SHH-03-043	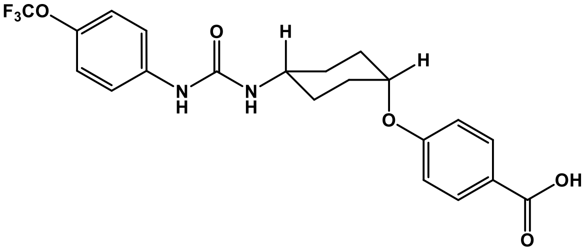	2.4	6.9	0.4
**1707**	SHH-03-062	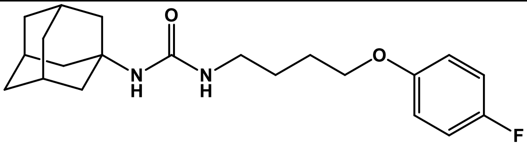	0.5	0.5	0.9
**1728**	SHH-03-078	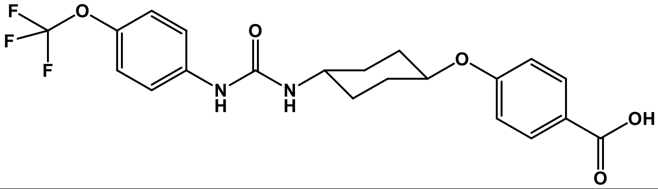	0.5	0.4	0.9
**1761**	SHH-04-002	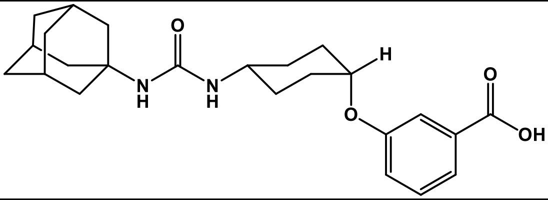	10.7	6.6	11.6

**1762**	SHH-03-097	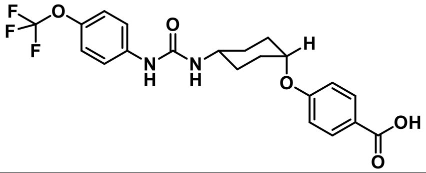	1.1	1.9	6.0
**1806**	JRS-PR-12	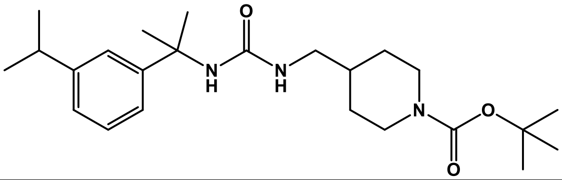	3.1	8.3	36.3
**1828**	LH-42	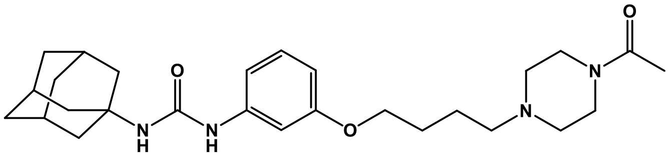	4.0	5.0	23.7
**1835**	LH-49	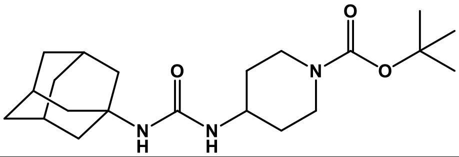	5.3	7.5	15.1
**1843**	TK-358-27-54	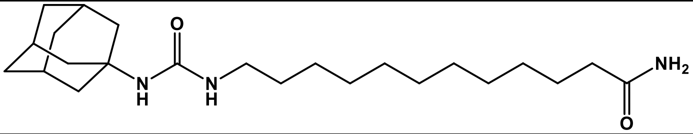	6.0	6.4	28.6
**1844**	TK-358-32-55	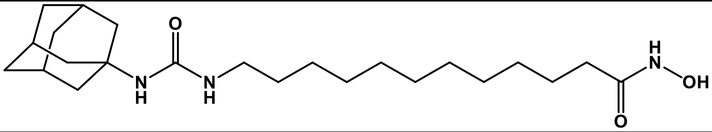	0.4	0.4	1.1
**1845**	TK-358-49-59	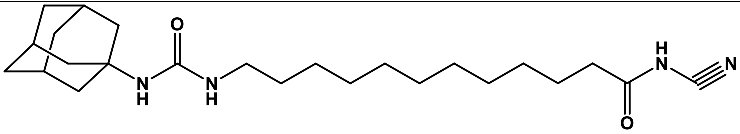	0.4	0.4	0.6
**1877**	HSH-34	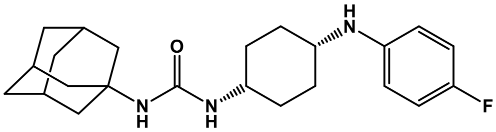	7.2	3.6	20.4
**2119**	SHH05082	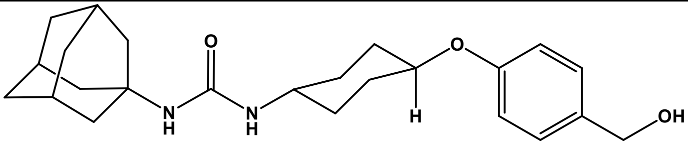	6.5	8.1	23.4
**2145**	BDH412-51A	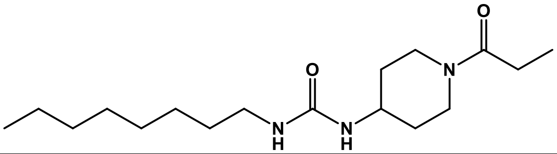	0.4	0.6	2.1
**2221**	SHH-06-071	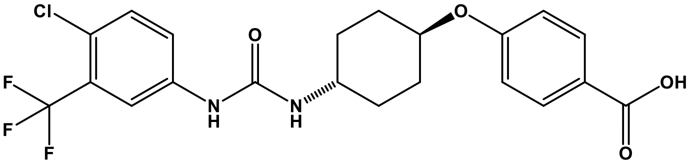	1.7	0.9	4.5
**2225**	SHH06094	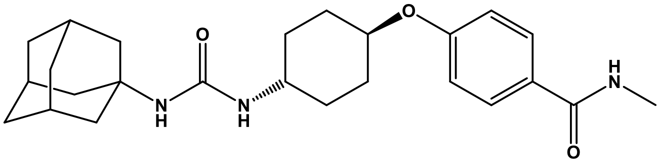	2.4	5.9	0.9

**2247**	SHH06091	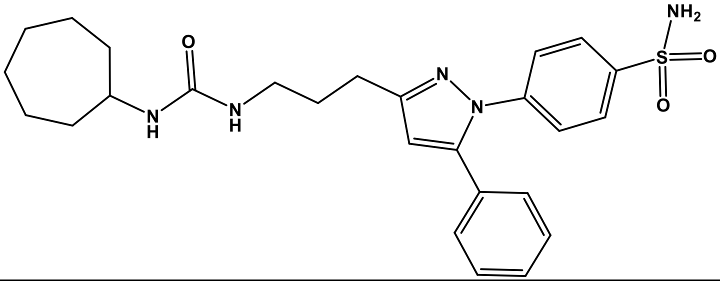	3.7	5.9	0.4
**2287**	SHH08002	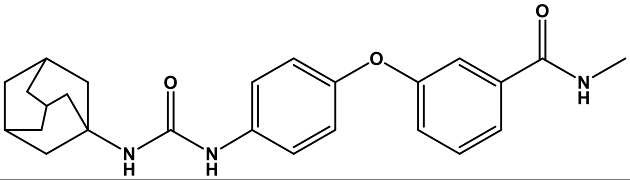	8.4	12.0	0.6
**2447**	SP-I-95C	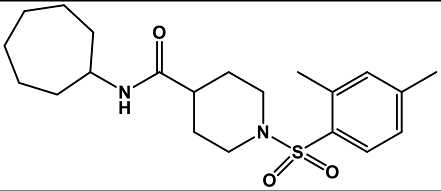	5.4	19.7	0.9
**2593**	SP-II-59C	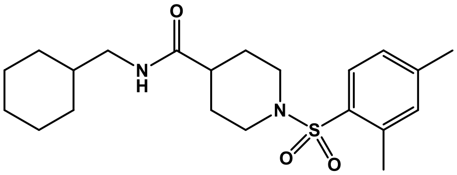	3.6	11.9	0.5
**2595**	SP-II-61C	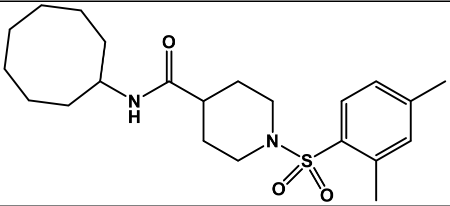	4.9	9.1	6.6
**2806**	SHH-09-074	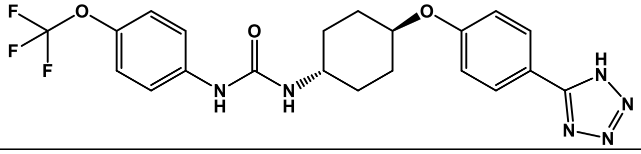	1.1	1.0	6.0
**2901**	SHH Syn 051	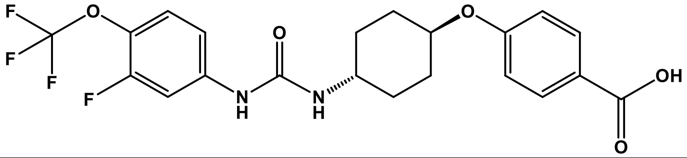	3.1	1.4	6.6
**2904**	SHH Syn 052	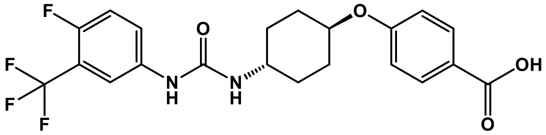	3.4	0.9	4.3
